# Myofibroblast‐Derived Extracellular Vesicles Drive Profibrotic Cascade Amplification in Pulmonary Fibrosis via the Nestin‐Rab7 Axis

**DOI:** 10.1002/jev2.70223

**Published:** 2026-01-06

**Authors:** Xiaofan Lai, Yong Xiao, Yingying Lin, Senyu Yao, Bin Wang, Hainan Chen, Tianxiang Lei, Shaojie Huang, Chenxing Lei, Qihao Zeng, Yuan Qiu, Hong Chen, Tao Wang, Jiancheng Wang, Andy Peng Xiang

**Affiliations:** ^1^ Department of Anesthesiology, The First Affiliated Hospital Sun Yat‐sen University Guangzhou China; ^2^ Center for Stem Cell Biology and Tissue Engineering, Key Laboratory for Stem Cells and Tissue Engineering, Ministry of Education Sun Yat‐Sen University Guangzhou China; ^3^ National‐Local Joint Engineering Research Center for Stem Cells and Regenerative Medicine, Zhongshan School of Medicine Sun Yat‐Sen University Guangzhou Guangdong China; ^4^ Department of Spine Surgery The Third Affiliated Hospital of Sun Yat‐Sen University Guangzhou China; ^5^ Department of Hematology, The Seventh Affiliated Hospital Sun Yat‐sen University Shenzhen Guangdong China; ^6^ Stem Cells and Regenerative Medicine Joint Laboratory of Sun Yat‐sen University and Gaozhou People's Hospital Sun Yat‐sen University Guangzhou China; ^7^ Department of Thyroid and Hernia Surgery, Guangdong Provincial People's Hospital Southern Medical University Guangzhou China; ^8^ Shenzhen Qianhai Shekou Free Trade Zone Hospital Shenzhen China; ^9^ Scientific Research Center The Seventh Affiliated Hospital of Sun Yat‐sen University Shenzhen China; ^10^ Department of Histoembryology and Cell Biology, Zhongshan School of Medicine Sun Yat‐Sen University Guangzhou Guangdong China

## Abstract

Idiopathic pulmonary fibrosis (IPF) is a fatal fibrotic lung disease characterized by aberrant myofibroblast activation and excessive extracellular matrix deposition, with extracellular vesicles (EVs) playing a crucial role in this pathological process. We observed that EVs levels are significantly elevated in IPF and positively correlate with nestin expression, a known marker of lung myofibroblasts. These myofibroblast‐derived EVs further amplify profibrotic responses, creating a self‐perpetuating cycle. To elucidate the mechanisms driving increased EVs secretion, we conducted in vitro and in vivo experiments, demonstrating that nestin knockdown not only suppresses EVs release but also impairs their ability to promote TGF‐β‐induced myofibroblast differentiation. Mechanistically, nestin recruits TBC1D15 to inactivate Rab7, thereby inhibiting multivesicular body (MVB) degradation and enhancing EVs secretion. Importantly, pharmacological activation of Rab7 using ML‐098 significantly attenuated pulmonary fibrosis in mouse models. Our findings establish the Nestin‐Rab7 axis as a key regulator of EVs‐mediated fibrotic signaling and highlight its therapeutic potential for IPF treatment.

## 1 Introduction

Idiopathic pulmonary fibrosis (IPF) is a chronic, progressive, and fatal interstitial lung disease characterized by high mortality and poor prognosis (Martinez et al. 2017). The disease manifests pathologically through the formation of myofibroblast foci and excessive deposition of extracellular matrix (ECM) in lung parenchyma, ultimately leading to respiratory failure with a median survival of only 2–3 years post‐diagnosis (Sgalla et al. 2016). Despite recent advances, current therapeutic options remain limited to slowing disease progression rather than achieving remission, largely due to incomplete understanding of its pathogenesis (Spagnolo et al. 2015). Central to IPF development are activated myofibroblasts, which serve as the primary effector cells responsible for pathological collagen deposition and fibrotic tissue remodeling (King et al. 2011). Elucidating the molecular mechanisms driving myofibroblast activation and persistence therefore represents a critical avenue for developing more effective therapeutic interventions against this devastating disease.

Extracellular vesicles (EVs) are nanoscale lipid‐bound particles released by virtually all cell types, serving as critical mediators of intercellular communication and disease pathogenesis (Zaborowski et al. 2015, Harding et al. 2013). These vesicles originate either through the budding of the plasma membrane or via the endosomal pathway, giving rise to distinct EV subpopulations (Hirsova et al. 2016, Mathieu et al. 2019). EVs are generated through the inward budding of late endosomes, forming multivesicular bodies (MVBs) containing intraluminal vesicles (ILVs) (Mathieu et al. 2019, Pegtel and Gould 2019, Kalluri and Lebleu 2020). This biogenesis process is regulated by both ESCRT (endosomal sorting complexes required for transport)‐dependent and ESCRT‐independent mechanisms (Vietri et al. 2020). MVBs subsequently undergo one of two fates: lysosomal degradation or fusion with the plasma membrane to release EVs into the extracellular space (Teng and Fussenegger 2020). Emerging evidence highlights the pathogenic role of EVs in IPF. Elevated EVs levels have been detected in bronchoalveolar lavage fluid (BALF) from both experimental fibrosis models and IPF patients, these EVs carry profibrotic cargo, including WNT5A, which actively promotes disease progression (Martin‐Medina et al. 2018). Notably, lung myofibroblast‐derived EVs are particularly abundant in the IPF lung microenvironment, where they are postulated to amplify fibrotic signaling (Burgy et al. 2024, Kadota et al. 2020). However, the precise molecular mechanisms driving their increased production and secretion remain incompletely understood, representing a critical knowledge gap in IPF pathogenesis.

Abundant evidence suggests that the Rab family of small GTPases plays a critical role in EVs trafficking and release (Hsu et al. 2010, Ostrowski et al. 2010). For instance, Rab5 is primarily localized on early endosomes and is involved in MVB formation (Simonsen et al. 1998, Stenmark 2009). The late endosome‐associated GTP‐Rab7 regulates lysosomal targeting of MVBs (Rocha et al. 2009). Rab11 is also closely related to the release of EVs from cells. In addition, Rab27 mainly regulates the MVB morphology and docking to the plasma membrane for EVs release (Ostrowski et al. 2010). Nestin, a member of the class VI intermediate filament protein family, was originally identified as a marker for neural stem/progenitor cells of the developing central nervous system (Hockfield and Mckay 1985, Lendahl et al. 1990). Abundant evidence suggests that nestin also plays critical roles in tissue injury and regeneration across various tissues and organs (Jiang et al. 2014, Méndez‐Ferrer et al. 2010). Our previous studies proved that nestin was markedly upregulated and facilitated Rab11‐dependent recycling of TβRI in experimental pulmonary fibrosis and IPF patients (Wang et al. 2022). Thus, we aim to explore whether increased EVs levels from myofibroblasts in pulmonary fibrosis were related with nestin and the Rab family in the present study. We clarified the molecular mechanisms of EVs trafficking in pulmonary fibrosis, thereby amplifying profibrotic responses in the lung microenvironment.

## 2 Material and Methods

### 2.1 Animal Experiments

Male C57BL/6 mice (8–12 weeks old) were provided by the Sun Yat‐sen University Animal Center (Guangzhou, China). Mice were randomly allocated to each group before experiments. All the researchers were blinded to the group arrangement. For the bleomycin‐induced pulmonary fibrosis model, mice were anesthetized with isoflurane and intratracheally injected with 3 U/kg bleomycin in 50 µL saline solution or an equal volume of PBS. Lung samples were harvested at 21 days for analysis. All the mice were allowed free access to standard food and water and housed in ventilated cages on a 12 h light/dark cycle at 22°C –24°C and 40%–60% humidity. All animal procedures were performed in accordance with the protocols approved by the Ethics Committee of Sun Yat‐sen University (Guangzhou, China).

### 2.2 Patient Samples

Human lung tissues with fibrosis were obtained by diagnostic surgical lung biopsies from patients who fulfilled the diagnostic criteria for IPF at the First Affiliated Hospital of Guangzhou Medical University, Guangzhou, China. Control human lung tissue samples were collected from surgical lung resection from patients with lung cancer at the same hospital. All patients signed informed consent for their samples to be used for research, and approval was obtained from the Committees for Ethical Review of Research.

### 2.3 Cell Culture

Primary mouse lung fibroblast isolation was performed as previously described (Weng et al. 2019). Fresh lungs from 12‐week‐old normal mice were perfused, isolated under sterile conditions, and excised into approximately 1‐mm^3^ fragments. These fragments were digested in DMEM supplemented with 10 mg/mL dispase (Sigma‐Aldrich, D4693) and 20 mg/mL collagenase type I (Gibco, Thermo Fisher Scientific, 9001‐12‐1) at 37°C for 2 h, followed by sequential filtration through 100‐µm, 40‐µm, and 15‐µm pore filters to remove undigested tissue debris. Single cells were washed to eliminate residual enzymes and maintained for 1 week in DMEM containing 10% fetal bovine serum (FBS, Gibco) and penicillin‐streptomycin. The cells were then cultured in a humidified 5% CO_2_ atmosphere at 37°C, as previously described, to facilitate fibroblast growth and dominance (Eickelberg et al. 2001). Once the outgrowing fibroblasts reached confluence, they were passaged via trypsinization. Fibroblasts used for experiments were between passages 3 and 6. Identification of fibroblasts was confirmed by the expression of vimentin, collagen I, and α‐SMA. Human fetal pulmonary fibroblasts (MRC5 cells) and HEK293T cells were purchased from the American Type Culture Collection (ATCC) and were cultured in DMEM medium containing 10% FBS and 1% penicillin‐streptomycin in a humidified incubator of 5% CO2 at 37°C.

### 2.4 Vectors and Reagents

For knockdown of nestin expression, retrovirus vectors (pSM2) encoding shRNA were purchased from Open Biosystems (Huntsville, AL, USA) and used as previously described (Wang et al. 2022). Scramble shRNA served as a control. Details on the plasmids were listed in Table S1. The retrovirus vectors encoding TBC1D15 shRNA were purchased from the Hanbio Biotechnology Co., Ltd. (Shanghai, China). shRNA transfections were conducted using lipo8000 transfection reagent (Beyotime, China) following the manufacturer's instructions. TGF‐β was purchased from Sigma‐Aldrich (USA).

### 2.5 Real‐time Quantitative PCR

Total RNA was extracted from tissues or cells with TRIzol reagent (Molecular Research Center, Inc., USA) according to the manufacturer's protocol. Complementary DNA (cDNA) was synthesized with a RevertAid First Strand cDNA Synthesis Kit (Thermo Fisher Scientific, USA). The real‐time quantitative PCR (qPCR) reactions were performed in triplicate with the FastStart Essential DNA Green Master Mix (Roche, Germany). 18S rRNA or GAPDH was used to normalize the mRNA expression. The 2−ΔΔCT method was used to calculate Fluorescence Signals (FCs). The primers designed and used for qPCR were listed in Table S2.

### 2.6 EVs Isolation

Before isolating EVs from the cellular supernatant, cells were cultured in DMEM medium containing 10% exosome‐depleted FBS (SBI, USA). After 48 h of cell culture, the cell culture supernatants were collected, and then EVs were isolated at 4°C by serial centrifugation. The supernatants were pelleted at 3000× g for 15 min to remove cellular debris. Then the supernatants were collected and centrifuged at 10,000 × g for 30 min at 4°C to remove larger microvesicles and apoptotic bodies. The obtained supernatants were filtered through a 0.22 µm filter, and the EVs were pelleted by ultracentrifugation at 120,000 × g for 70 min using an ultracentrifuge (Beckman Coulter, Optima L‐100 XP, USA). The obtained pellet was resuspended in lysis buffer or PBS for analysis.

### 2.7 Transmission Electron Microscopy

The freshly isolated EVs were gently pelleted in Eppendorf tubes. The EVs in Eppendorf tubes were first dried on a formvar/carbon‐coated copper grid for 10 min and fixed in 3% glutaraldehyde for 10 min and then contrasted in a uranyl acetate (4%) / methylcellulose (1%) mix for 10 min. The samples were observed immediately using an electron microscope (JEOL, 1200EX, Japan), and the images were analyzed by ImageJ software version 1.50i (National Institutes of Health, USA).

### 2.8 Histopathological Evaluation

After sacrifice, mouse lung tissues were fixed with 4% paraformaldehyde and then embedded in paraffin. Paraffin‐embedded lung tissue sections were sectioned into 5 µm thick slices and placed on glass slides. Following deparaffinization, tissue samples were exposed to hematoxylin and eosin (H&E) staining to analyze the structure of lungs. Masson's trichrome staining was performed using Masson's trichrome staining kit according to the manufacturer’ instructions (Thermo Fisher Scientific, USA). The fibrosis degree of lung samples was assessed using ImageJ software.

### 2.9 Immunofluorescence Staining

The lung tissue sections after deparaffinization and antigen retrieval or cells grown on glass coverslips were incubated overnight at 4°C with corresponding primary antibodies. After washing in PBS three times, the samples were incubated with corresponding secondary antibodies for 1 h. After washing in PBS three times, nuclei of cells were stained with DAPI dye (Roche, USA). Images were captured with a Zeiss 800 Laser Scanning Confocal Microscope and a Zeiss 880 Laser Scanning Confocal Microscope with Airyscan (Zeiss, Germany). ZEN software (Zeiss, Germany) and ImageJ software were used for image processing and analysis. The primary and secondary antibodies used were listed in Table S3.

### 2.10 Adeno‐Associated Virus (AAV) Delivery

The adeno‐associated virus vector serotype 6 (AAV6) expressing nestin shRNA or scramble shRNA was purchased from Hanbio Biotechnology Co., Ltd. (Shanghai, China). The C57BL/6 mice (8–12 weeks old) were anesthetized with isoflurane and intratracheally injected with 1.5×10^12^ viral genomes of AAV6 scramble shRNA vector or 1.5×10^12^ viral genomes of AAV6 nestin shRNA vector.

### 2.11 Western Blot and Co‐Immunoprecipitation (Co‐IP)

Total protein lysates were lysed with RIPA buffer (Thermo Fisher Scientific, USA) following the manufacturer's instructions. The proteins were separated by sodium dodecyl sulfate‐polyacrylamide gel electrophoresis (SDS‐PAGE, Bio‐rad Laboratories, USA) and transferred to polyvinylidene fluoride (PVDF) membranes (Millipore, USA). The proteins were probed with corresponding primary antibodies overnight at 4°C followed by HRP‐conjugated secondary antibodies for 1 h. After washing three times, the membranes were covered with Immobilon Western Chemiluminescent HRP substrate (Millipore, USA) and imaged using the ChemiDoc XRS+ System (Bio‐Rad Laboratories, USA). Co‐immunoprecipitation (Co‐IP) was conducted using the Pierce Co‐Immunoprecipitation Kit (Thermo Fisher Scientific, USA) as previously described (Wang et al. 2022). Finally, the proteins were processed for western blot, and bands were quantified using ImageJ software. All the antibodies were listed in Table S3.

### 2.12 Rab7 Pull‐Down Activation Assay

The Rab7 activation was assessed using a Rab7 activation assay kit (NewEast Biosciences, USA) according to the manufacturer's instructions. In brief, cell lysates containing Rab7‐GTP were first incubated with anti‐Rab7‐GTP mouse monoclonal antibody overnight at 4°C. The bound active Rab7 was pulled down with protein A/G agarose for 1 h at 4°C and detected through immunoblot analysis using anti‐Rab7 rabbit polyclonal antibody.

### 2.13 Hydroxyproline Assay

Mouse lung tissue samples were homogenized with trichloroacetic acid and incubated in 250 µL of 12 M HCl at 110°C overnight. Then samples were neutralized by adding 10 M NaOH. The hydroxyproline content was measured by use of chloramine‐T as previously described (Wang et al. 2022). Hydroxyproline levels were expressed as micrograms of hydroxyproline per microgram of lung samples.

### 2.14 Statistical Analysis

All data were reported as the mean ± SD of at least three independent experiments. The sample sizes per group are all presented in the figure legends. Sample comparison between two groups was assessed using an unpaired *t*‐test. Sample comparison between multiple groups was performed by one‐way ANOVA, with Tukey's multiple comparison test. Statistical significance was analyzed using GraphPad Prism (version 8.0.1). Statistical significance was taken as *p *< 0.05, with significance defined as *p *< 0.05, *p *< 0.01, and *p *< 0.001.

## 3 Results

### 3.1 Myofibroblast‐Derived EVs Drive Profibrotic Cascade Amplification in Pulmonary Fibrosis

**FIGURE 1:**
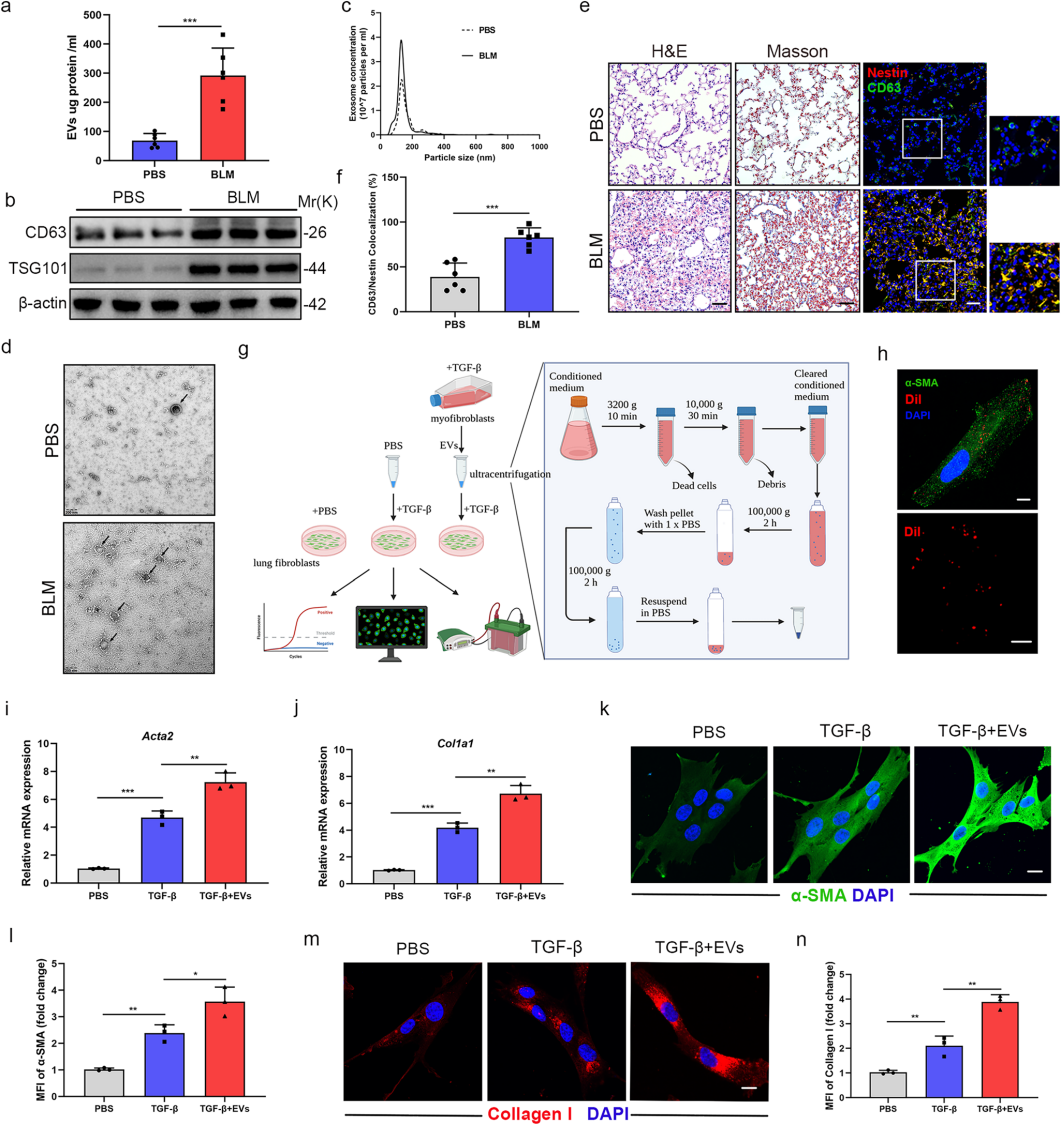
**Myofibroblast‐derived EVs drive profibrotic cascade amplification in pulmonary fibrosis**. (a) Concentrations of EVs proteins in the bronchoalveolar lavage fluid (BALF) from mice isolated by sequential ultracentrifugation (*n* = 6 per group). (b) Western blot analysis of CD63 and TSG101 expression in BALF from mice 21 days after bleomycin exposure. (c) Representative particle size and concentration distribution of EVs purified from BALF of mice isolated by sequential ultracentrifugation cell culture supernatants by nanoparticle tracking analysis (NTA). (d) Representative electron microscopic images of EVs purified from BALF of mice isolated by sequential ultracentrifugation cell culture supernatants. Scale bar = 200 nm. (e) Hematoxylin and eosin staining, Masson's trichrome staining and immunofluorescence images obtained using anti‐nestin (red), anti‐CD63 (green) antibody of lung sections from C57/BL6 mice 21 days after bleomycin exposure. Scale bars: 100 µm. (f) Quantification analysis of CD63 and nestin colocalization of lung sections from C57/BL6 mice 21 days after bleomycin exposure from (e) (*n* = 6 per group). (g) Schematic overview of experimental design. Primary mouse lung fibroblasts were treated with TGF‐β (5 ng mL^−1^) for 24 h. After being washed by DMEM medium, cells were cultured in DMEM medium for 48 h and then the EVs were isolated by ultracentrifugation from their conditioned medium. Then we exposed primary mouse lung fibroblasts to obtained EVs or PBS with TGF‐β (5 ng mL^−1^) in different groups for 72 h and collected cells for analysis. Created in https://BioRender.com. (h) Immunofluorescence staining of α‐SMA (green) and dil (red) in primary mouse lung myofibroblasts treated with Dil‐labeled EVs. Control image shows Dil‐labeled EVs in PBS. Scale bars = 20 µm. (i) qPCR analysis of Acta2 mRNA expression in primary mouse lung fibroblasts treated with or without EVs in different groups for 72 h (*n* = 3). (j) qPCR analysis of col1a1 mRNA expression in primary mouse lung fibroblasts treated with or without EVs in different groups for 72 h (*n* = 3). (k) Immunofluorescence staining and (l) quantification analysis of primary mouse lung fibroblasts treated with or without EVs in different groups for 72 h using anti‐α‐SMA (green) antibody. Scale bars: 20 µm. (m) Immunofluorescence staining and (n) quantification analysis of primary mouse lung fibroblasts treated with or without EVs in different groups for 72 h using anti‐collagen I (red) antibody. Scale bars: 20 µm. Data are presented as the mean ± SD of three independent experiments; *p < 0.05, **p < 0.01, ***p < 0.001; one‐way ANOVA and Tukey's multiple comparisons test.

We first established a bleomycin‐induced pulmonary fibrosis murine model as previously described (Wang et al. 2022) to investigate EVs dynamics in fibrotic lungs. There was severe inflammatory infiltration, interstitial collagen deposition, and fibrosis degree in lung samples from bleomycin‐treated mice, as indicated by H&E and Masson's trichrome staining (Figure 1e and Figure S1c). Quantitative analysis of bronchoalveolar lavage fluid (BALF)‐derived EVs, isolated via sequential ultracentrifugation, revealed a significant increase in total EVs protein content in bleomycin‐treated mice compared to controls (Figure 1a). Western blot analysis confirmed upregulation of canonical EVs markers (CD63, TSG101) in bleomycin‐exposed BALF (Figure 1b and Figure S1a,b). Nanoparticle tracking analysis (NTA) and transmission electron microscopy demonstrated that ultracentrifugation pellets contained vesicles of typical EVs size (50–150 nm), with substantially greater abundance in the bleomycin group (Figure 1c,d). Notably, immunofluorescence co‐staining showed enhanced CD63 expression in fibrotic lungs, where it predominantly colocalized with Nestin—the class VI intermediate filament we previously identified as a lung myofibroblast marker (Figure 1e,f). Collectively, these findings identify the myofibroblast as a major source of pathogenic EVs in pulmonary fibrosis.

To functionally characterize these vesicles, we isolated EVs from lung myofibroblasts and treated primary mouse lung fibroblasts under TGF‐β stimulation for 72 h (Figure 1g). Confocal microscopy confirmed efficient internalization of Dil‐labeled EVs into recipient myofibroblasts, with predominant cytoplasmic localization (Figure 1h). qRT‐PCR revealed significantly elevated mRNA levels of α‐SMA (Acta2) and collagen I (Col1a1) in the TGF‐β + EVs group versus TGF‐β alone (Figure 1i,j). Immunofluorescence showed intensified α‐SMA stress fibers and collagen I deposition in EVs‐treated groups (Figure 1k–n). These results indicate lung myofibroblast‐derived EVs are elevated during fibrosis and promote cascade amplification of profibrotic responses.

### 3.2 Nestin Knockdown Decreased EVs Secretion In Vivo in Fibrotic Lungs

**FIGURE 2:**
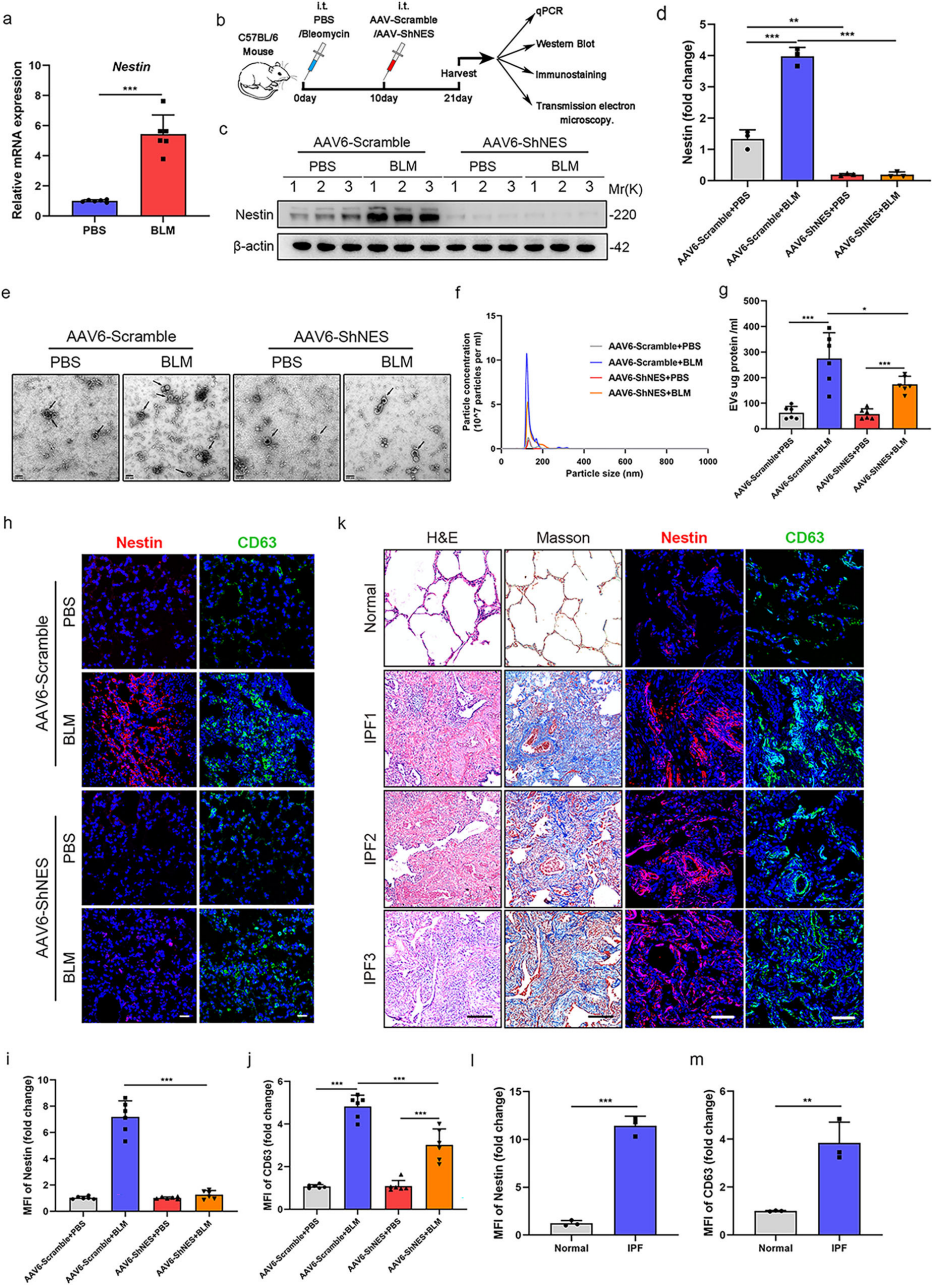
**Nestin knockdown decreased EVs secretion in vivo in fibrotic lungs**. (a) qPCR analysis of the nestin mRNA expression in lung sections from C57/BL6 mice 21 days after bleomycin exposure (*n* = 6 per group). (b) Experimental design. C57BL/6 mice were intratracheally injected with bleomycin (3U/kg) or PBS. After 10 days, the mice were intratracheally injected with AAV6‐ShNES or AAV6‐Scramble. After 21 days, lung samples were collected for analysis. Created in https://BioRender.com. (c) Western blot analysis and (d) quantification analysis of nestin expression in the lungs of C57/BL6 mice from the different groups (*n* = 3). (e) Representative electron microscopic images of EVs purified from cell culture supernatants. Scale bar = 200 nm. (f) Representative particle size and concentration distribution of EVs purified from BALF of mice isolated by sequential ultracentrifugation cell culture supernatants by nanoparticle tracking analysis (NTA). (g) Concentrations of EVs proteins in the BALF from mice isolated by sequential ultracentrifugation from the different groups (*n* = 6 mice per group). (h) Immunofluorescence images obtained using anti‐nestin (red), anti‐CD63 (green) antibody of lung sections from C57/BL6 mice in different groups (*n* = 6 per group) and quantification analysis of (i) Nestin and (j) CD63. Scale bars: 100 µm. (k) Hematoxylin and eosin staining, Masson's trichrome staining and immunofluorescence images obtained using anti‐Nestin (red), anti‐CD63 (green) antibody of lung sections from Idiopathic pulmonary fibrosis (IPF) patients and healthy donors and quantification analysis of (l) Nestin and (m) CD63. Scale bars: 200 µm. Data are presented as the mean ± SD of three independent experiments; *p < 0.05, **p < 0.01, ***p < 0.001; one‐way ANOVA and Tukey's multiple comparisons test.

Our previous studies have proved that nestin facilitated the recycling of TβRI in myofibroblasts in experimental pulmonary fibrosis and IPF patients, a process primarily regulated by Rab proteins (Wang et al. 2022). Given the established critical role of the Rab family in EVs trafficking and release (Hsu et al. 2010, Ostrowski et al. 2010), we hypothesized that nestin is also involved in EVs secretion from lung myofibroblasts. To determine whether nestin expression correlated with EVs release in pulmonary fibrosis, we first tested the expression of nestin and CD63 in mice lungs and found that they are both significantly increased in fibrotic lungs compared with those from control lungs (Figure 1e, Figure 2a and Figure S1d). Recently, AAV vectors have been regarded as an effective and relatively safe gene delivery tool in clinical trials due to their low oncogenicity and weak immunogenicity (Crystal 2014, Li et al. 2011). It was demonstrated that AAV6 exhibits improved transduction efficiency compared to previously reported AAVs in mouse airways and in culture models of human airway epithelium (Limberis et al. 2009). Thus, we established the bleomycin‐induced pulmonary fibrosis model in C57/BL6 mice and injected the Adeno‐associated virus serotype 6 (AAV6)‐Scramble or AAV6‐ShNES of equal volume intratracheally to the mice 10 days later in order to downregulate the nestin expression of myofibroblasts (Figure 2b). Twenty‐one days after bleomycin administration, nestin was successfully downregulated in the lungs from the AAV6‐ShNES group (Figure 2c,d). Interestingly, electron microscopic analysis and NTA analysis showed that pellets isolated by ultracentrifugation were significantly decreased in BALF from the AAV6‐ShNES + BLM group compared with those from the AAV6‐Scramble + PBS group (Figure 2e,f). Similarly, nestin downregulation inhibited the secretion of EVs proteins and reduced the levels of EVs markers CD63 and TSG101 in purified EVs from pulmonary fibrosis mice models (Figure 2g‐j), which indicated that nestin is positively correlated with EVs release in experimental pulmonary fibrosis. To further validate these observations, we analyzed the expression of nestin and CD63 in the lungs from IPF patients and healthy donors. Both nestin and CD63 were markedly increased in IPF fibrotic lungs compared with those from normal lungs (Figure 2k–m). The above data supported that nestin knockdown decreased EVs secretion in fibrotic lungs in vivo.

### 3.3 Nestin Knockdown Attenuates the Ability of EVs to Promote TGF‐β‐Induced Myofibroblast Differentiation

**FIGURE 3:**
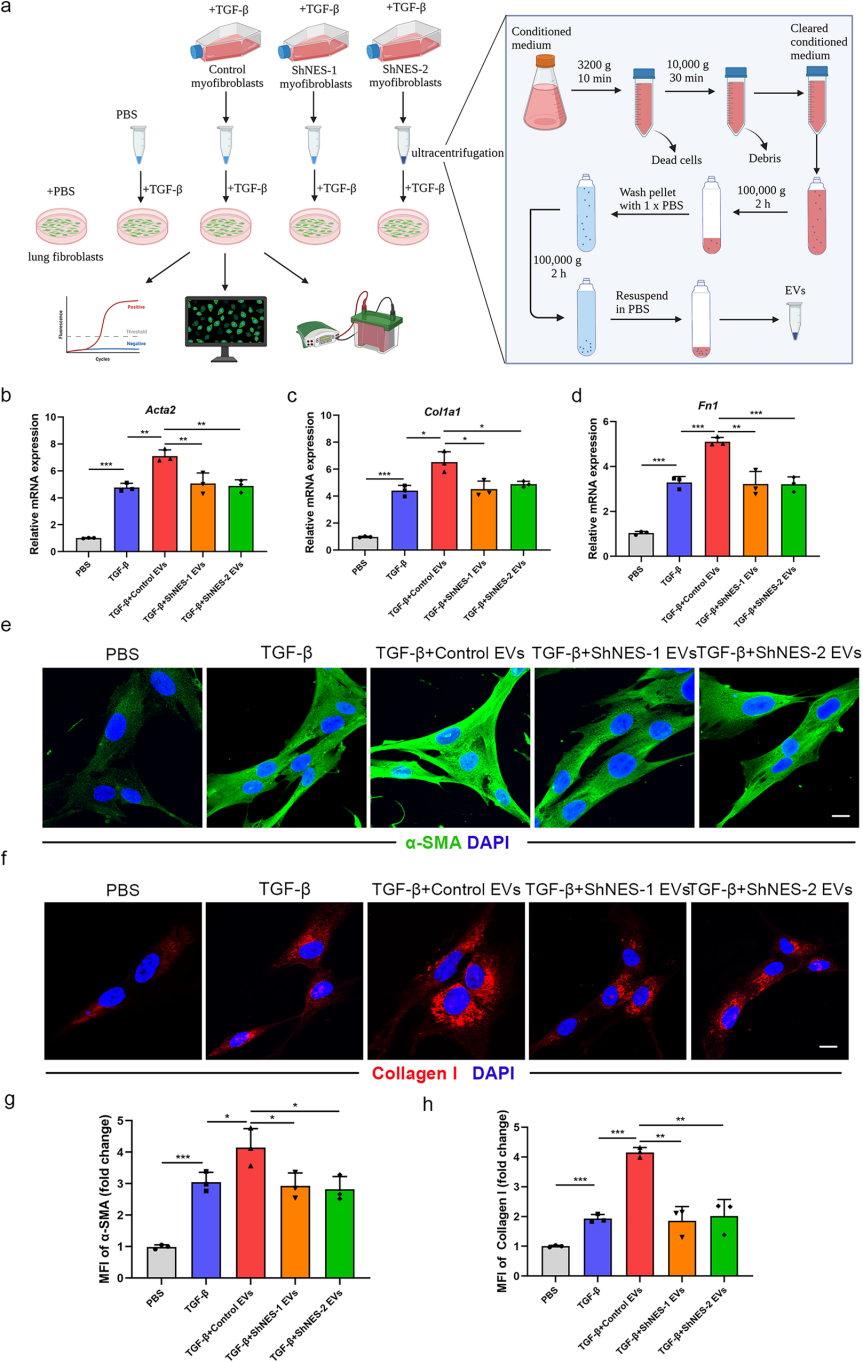
**Nestin knockdown attenuates the ability of EVs to promote TGF‐β‐induced myofibroblast differentiation**. (a) Schematic overview of the experimental design. Primary mouse lung fibroblasts were treated with TGF‐β (5 ng mL^−1^) for 24 h. After being washed by DMEM medium, cells were cultured in DMEM medium for 48 h and then the EVs were isolated by ultracentrifugation from their conditioned medium. Then we exposed primary mouse lung fibroblasts to obtained EVs with or without TGF‐β (5 ng mL^−1^) in different groups for 72 h and collected cells for analysis. Created in https://BioRender.com. (b) qPCR analysis of Acta2 mRNA expression in primary mouse lung fibroblasts treated with EVs in different groups for 72 h (*n* = 3). (c) qPCR analysis of Col1a1 mRNA expression in primary mouse lung fibroblasts treated with EVs in different groups for 72 h (*n* = 3). (d) qPCR analysis of Fn1 mRNA expression in primary mouse lung fibroblasts treated with EVs in different groups for 72 h (*n* = 3). Immunofluorescence staining of primary mouse lung fibroblasts treated with EVs in different groups for 72 h using (e) anti‐α‐SMA (green) antibody and (f) anti‐collagen I (red) antibody [Scale bars: 20 µm]. Quantification analyses of primary mouse lung fibroblasts treated with EVs in different groups for 72 h using (g) anti‐α‐SMA (green) antibody and (h) anti‐collagen I (red) antibody [Scale bars: 20 µm]. Data are presented as the mean ± SD of three independent experiments; *p < 0.05, **p < 0.01, ***p < 0.001; one‐way ANOVA and Tukey's multiple comparisons test.

We next investigated whether nestin influences the functional effects of EVs. Primary mouse lung fibroblasts were treated with EVs isolated from control or nestin‐knockdown cells with TGF‐β stimulation for 72 h (Figure 3a). Through qRT‐PCR analysis we found that the mRNA levels of α‐SMA, collagen I, and fibronectin increased in cells treated with EVs from control cells (TGF‐β + Control EVs group) compared to those treated with EVs from Nestin‐knockdown cells (TGF‐β + ShNES‐1/2 EVs groups) (Figure 3b–d). Immunofluorescence staining and western blot revealed that the α‐SMA and collagen I expression increased in the TGF‐β+Control EVs group but not in cells treated with EVs from Nestin‐knockdown cells compared with the TGF‐β group (Figure 3e–h and Figure S2d–f). In addition, we proved that EVs from the control group had a modest pro‐fibrotic effect on their own. However, this effect was significantly less pronounced than when EVs were combined with TGF‐β, which suggests that Nestin‐enhanced EVs act to amplify TGF‐β‐driven myofibroblast differentiation, rather than initiating it independently (Figure S2a–f). Thus, we conclude that nestin plays a critical role in regulating EVs release and therefore promoting myofibroblast activation.

### 3.4 Nestin Knockdown Inhibits EVs Secretion In Vitro

**FIGURE 4:**
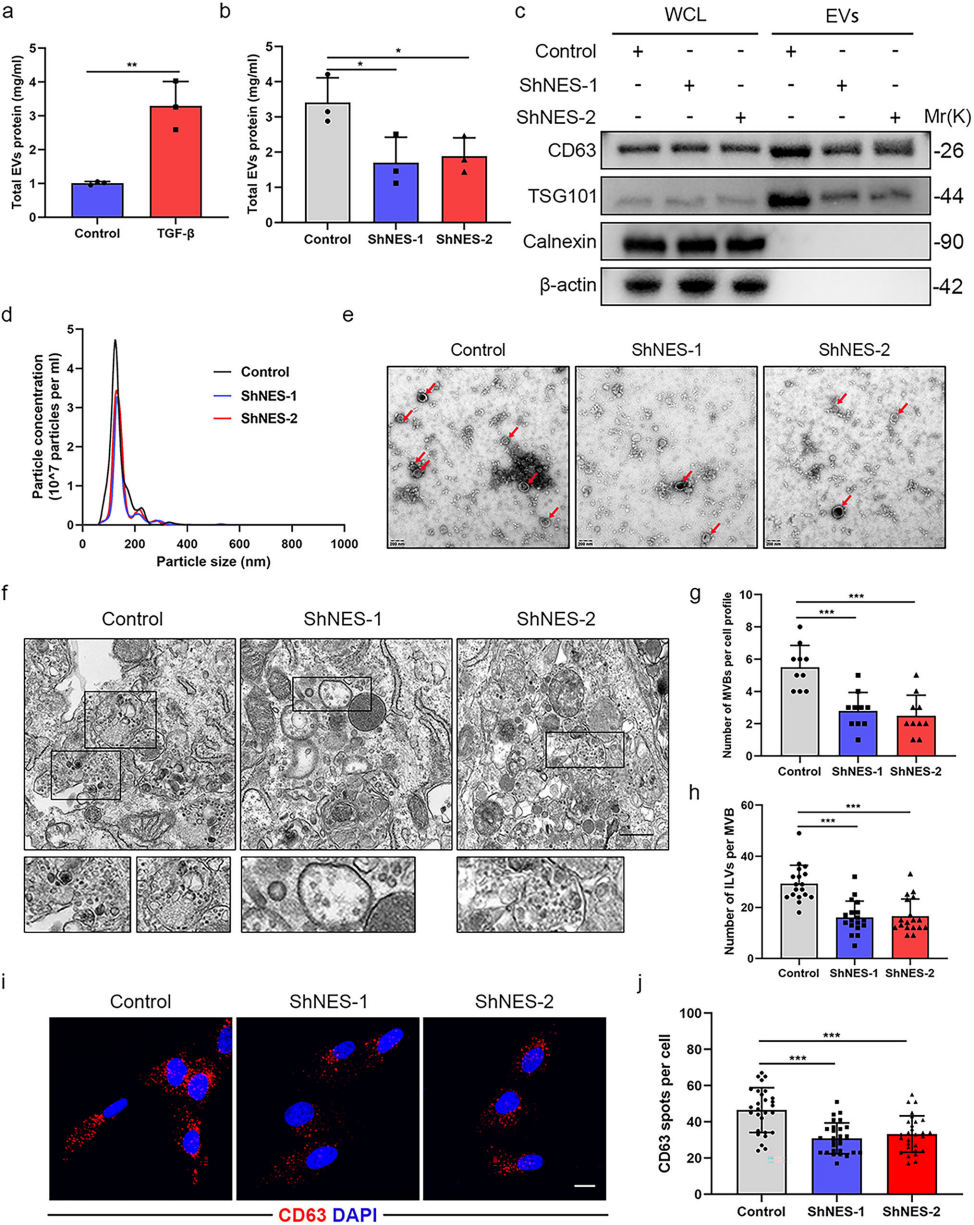
**Nestin knockdown inhibits EVs secretion in vitro**. (a) Concentrations of EVs proteins in cell culture supernatants from primary mouse lung fibroblasts treated with or without TGF‐β (5 ng mL^−1^) (*n* = 3). (b) Concentrations of EVs proteins in cell culture supernatants from Nestin‐knockdown cells and control cells treated with TGF‐β (5 ng mL^−1^) (*n* = 3). (c) Western blot in Whole cell lysates (WCL) and EVs purified from cell culture supernatants of Nestin‐knockdown cells and control cells treated with TGF‐β (5 ng mL^−1^). We used β‐actin as internal control and calculated the ratio of the gray value of CD63 and TSG101 to that of β‐actin to obtain their relative expression level. (d) Representative particle size and concentration distribution of EVs purified from cell culture supernatants of Nestin‐knockdown cells and control cells treated with TGF‐β (5 ng mL^−1^) by NTA. (e) Representative electron microscopic images of EVs purified from cell culture supernatants of Nestin‐knockdown cells and control cells treated with TGF‐β (5 ng mL^−1^). Scale bar = 200 nm. (f) Representative electron microscopic images of Nestin‐knockdown cells and control cells treated with TGF‐β (5 ng mL^−1^). Scale bar = 500 nm. (g) The number of MVBs per cell profile from Figure 2f. (h) The number of ILVs per MVB from Figure 2f. (i) Immunofluorescence staining and (j) quantification analysis of Nestin‐knockdown cells and control cells treated with TGF‐β (5 ng mL^−1^) using anti‐CD63 (red) antibody. Scale bars: 20 µm. Data are presented as the mean ± SD of three independent experiments; *p < 0.05, **p < 0.01, ***p < 0.001; One‐way ANOVA and Tukey's multiple comparisons test.

To gain further insight into the mechanisms by which nestin regulates EVs secretion, we first induced primary mouse lung fibroblasts to differentiate into myofibroblasts by TGF‐β stimulation and found that total EVs protein levels were markedly increased in myofibroblasts relative to control (Figure 4a). Then we knockdown the expression of nestin by two different short hairpin RNAs as previously described in primary mouse lung fibroblasts and performed in vitro analysis. We found that nestin knockdown resulted in a significant reduction in total EVs protein secretion (Figure 4b). The EVs markers CD63 and TSG101 were both decreased in EVs isolated from the conditioned media of Nestin‐knockdown cells relatively (Figure 4c and Figure S3a,b). NTA and electron microscopic analysis confirmed that the size and morphology of EVs were unaffected by nestin knockdown (Figure 4d,e). Besides, we also adopted the cell line human embryonic lung fibroblasts (MRC5) for analysis and proved that the total EVs protein was significantly decreased after nestin knockdown (Figure S3c). NTA and electron microscopic analysis showed that the size and morphology of EVs were identical in MRC5 of different groups, while EVs concentration was significantly decreased in Nestin‐knockdown cells relative to control (Figure S3d,e). These results demonstrated that nestin knockdown inhibits EVs secretion in vitro without affecting their morphology and size.

It has been reported that EVs biogenesis begins with the invagination of late endosomes, leading to the formation of MVBs containing ILVs (Vietri et al. 2020). To investigate how nestin regulates EVs secretion, we examined the number and morphology of MVBs and ILVs using electron microscopy. Surprisingly, we observed that nestin knockdown significantly reduced the number of MVBs per cell and the number of ILVs per MVB (Figure 4f–h). Similar results were observed in MRC5s (Figure S2f–h).

In addition, immunofluorescence imaging also revealed a decrease in CD63‐positive spots per cell in Nestin‐knockdown cells compared to controls (Figures 4i,j and S3i,j). These data suggested that nestin knockdown decreased the number of MVB per cell, thereby inhibiting EVs secretion.

### 3.5 Nestin Knockdown Inhibits EVs Secretion by Regulating Rab7 Activity

The Rab family of small GTPases plays a critical role in EVs trafficking and release (Hsu et al. 2010, Ostrowski et al. 2010, Hsu et al. 2010). For instance, Rab5 is primarily localized on early endosomes and is involved in MVB formation (Simonsen et al. 1998, Stenmark 2009). The late endosome associated GTP‐Rab7 regulates lysosomal targeting of MVBs (Rocha et al. 2009). Rab11 is also closely related with the release of EVs from cells. In addition, Rab27 mainly regulates the MVB morphology and docking to the plasma membrane for EVs release (Ostrowski et al. 2010). To determine whether nestin regulates EVs secretion through Rab GTPases, we first examined the expression of Rab5, Rab7, and Rab27 in Nestin‐knockdown cells. Western blot analysis revealed no significant differences in the protein levels of Rab5, Rab7, or Rab27 between Nestin‐knockdown and control cells (Figure S4a–d). Then we detected the co‐localization of Rab5 or Rab 11 with endogenous CD63, the well‐known marker of MVBs, in Nestin‐knockdown cells was identical to that in control cells (Figure S5a–d). Besides, CD63 colocalized more with Rab7+ vesicles but less with Rab27+ vesicles in Nestin‐knockdown cells relative to control (Figure S5e–h), which indicated that nestin might promote EVs secretion by regulating Rab7 or Rab27 activity.

**FIGURE 5:**
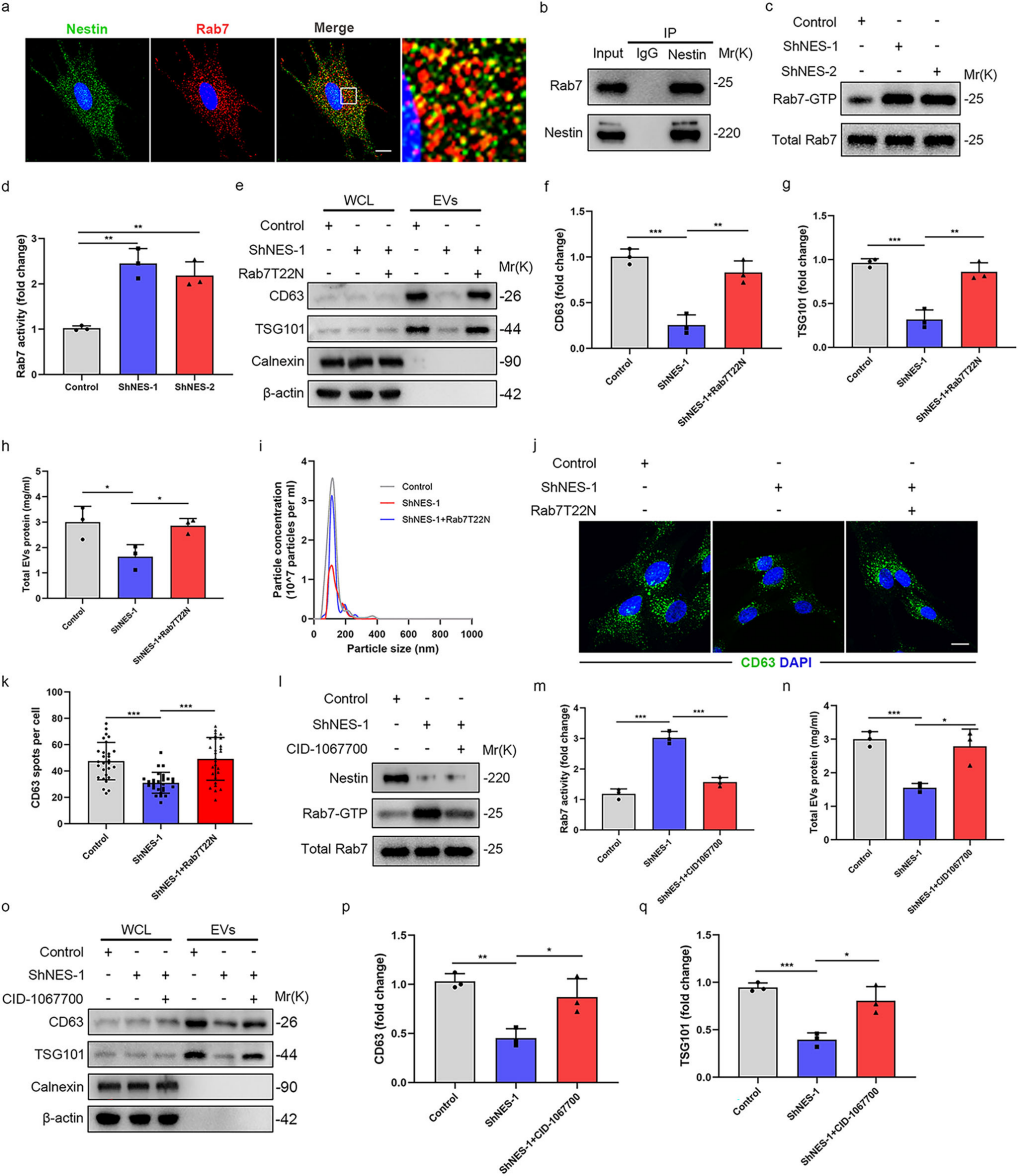
**Nestin knockdown inhibits EVs secretion by regulating Rab7 activity**. (a) Immunofluorescence staining of primary mouse lung fibroblasts using anti‐Rab7 (red) and anti‐Nestin (green) antibody. Scale bars: 10 µm. (b) Immunoprecipitation was performed using an anti‐Nestin antibody, and immunoblotting of the protein levels of Rab7 in primary mouse lung fibroblasts. (c) Rab7 activity assays and (d) quantification analysis were performed in Nestin‐knockdown cells and control cells (*n* = 3). (e) Western blot and (f, g) quantification analysis of CD63 and TSG101 expression in WCL and EVs purified by serial ultracentrifugation from cell culture supernatants of Nestin‐knockdown cells and control cells transfected with or without Rab7T22N (*n* = 3). (h) Concentrations of EVs proteins purified by serial ultracentrifugation from cell culture supernatants from equal numbers of Nestin‐knockdown cells and control cells transfected with or without Rab7T22N (*n* = 3). (i) Representative particle size and concentration distribution of EVs purified by serial ultracentrifugation from cell culture supernatants from equal numbers of Nestin‐knockdown cells and control cells transfected with or without Rab7T22N by NTA. (j) Immunofluorescence staining and (k) quantification analysis of Nestin‐knockdown and control cells using anti‐CD63 (green) antibody transfected with or without Rab7T22N. Scale bars: 20 µm. (l) Rab7 activity assays and (m) quantification analysis were performed in Nestin‐knockdown cells and control cells treated with or without CID‐1067700 (*n* = 3). (n) Concentrations of EVs proteins purified by serial ultracentrifugation from cell culture supernatants from equal numbers of Nestin‐knockdown cells and control cells treated with or without CID‐1067700 (*n* = 3). (o) Western blot and (p, q) quantification analysis of CD63 and TSG101 expression in WCL and EVs purified by serial ultracentrifugation from cell culture supernatants from equal numbers of Nestin‐knockdown cells and control cells treated with or without CID‐1067700 (*n* = 3). Data are presented as the mean ± SD of three independent experiments; *p < 0.05, **p < 0.01, ***p < 0.001; One‐way ANOVA and Tukey's multiple comparisons test.

Surprisingly, immunofluorescence and co‐immunoprecipitation experiments revealed that nestin strongly colocalizes with Rab7, but not Rab27 or Rab5 (Figure 5a,b and Figure S5i,j). Moreover, nestin knockdown facilitated Rab7 GTPase activity (Figure 5c,d). To ascertain whether nestin knockdown inhibits EVs secretion by regulating Rab7 activity, we transfected cells with Rab7T22N mutant, a dominant‐negative form of Rab7 GTPases that (Feng et al. 1995, Vitelli et al. 1997) inhibits endosome–lysosome fusion (Bucci et al. 2000). The results showed that Rab7T22N transfection largely restored EVs secretion in Nestin‐knockdown cells (Figure 5e–k). Moreover, we also adopted the Rab7 GTPase competitive inhibitor CID‐1067700 (Ghosh et al. 2020, Zeng et al. 2021) and proved that Rab7 activity was markedly inhibited after adding CID‐1067700 (Figure 5l,m). Treatment with the CID‐1067700 significantly rescued EVs secretion in Nestin‐knockdown cells (Figure 5m,q). Consistently, the results showed that Rab7 knockdown significantly promoted the activation and differentiation of Nestin‐knockdown myofibroblasts after TGF‐β treatment (Figure S6a–e). Altogether, these data support that nestin knockdown inhibits EVs secretion in vitro primarily by regulating Rab7 activity.

### 3.6 Nestin Recruits TBC1D15 to Inactivate Rab7

**FIGURE 6:**
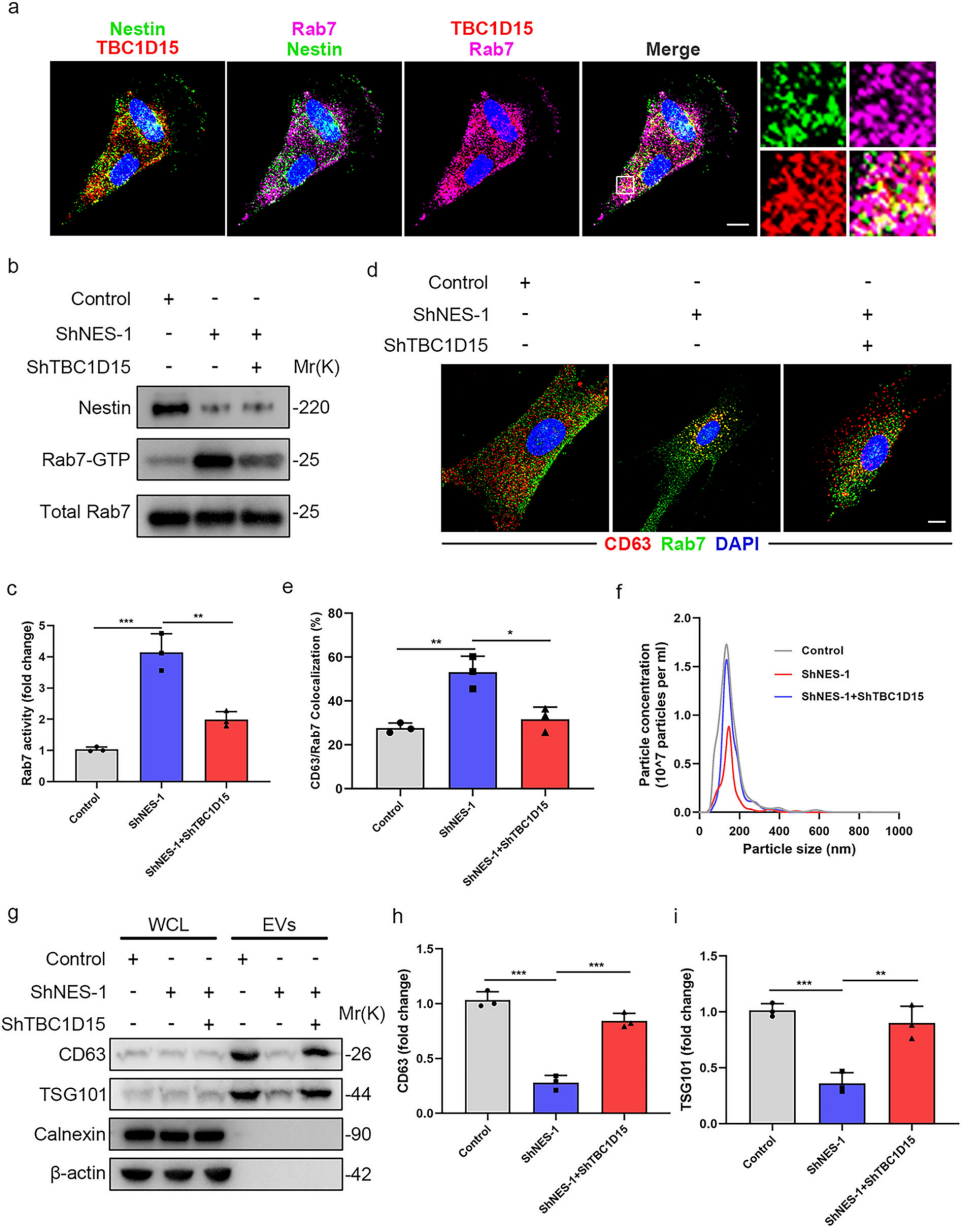
**Nestin recruits TBC1D15 to inactivate Rab7**. (a) Immunofluorescence staining of primary mouse lung fibroblasts using anti‐Rab7, anti‐TBC1D15 and anti‐Nestin antibody. Scale bars: 10 µm. (b) Rab7 activity assays and (c) quantification analysis were performed in primary mouse lung fibroblasts with nestin knockdown and TBC1D15 knockdown (*n* = 3). (d) Immunofluorescence staining and (e) colocalization analysis of primary mouse lung fibroblasts with nestin knockdown and TBC1D15 knockdown using anti‐CD63 (red) and anti‐Rab7 (green) antibody. Scale bars: 10 µm. (f) Representative particle size and concentration distribution of EVs purified by serial ultracentrifugation from cell culture supernatants from equal numbers of Nestin‐knockdown cells and control cells with or without TBC1D15 knockdown by NTA. (g) Western blot and (h, i) quantification analysis of CD63 and TSG101 expression in WCL and EVs purified by serial ultracentrifugation from cell culture supernatants from equal numbers of primary mouse lung fibroblasts with nestin knockdown and TBC1D15 knockdown (*n* = 3). Data are presented as the mean ± SD of three independent experiments; *p < 0.05, **p < 0.01, ***p < 0.001; One‐way ANOVA and Tukey's multiple comparisons test.

Rab7 cycles between an ‘active’ GTP‐bound form and an ‘inactive’ GDP‐bound form, which is regulated by several accessory proteins, including guanine nucleotide exchange factors (GEFs) for Rab7 activation and GTPase activating proteins (GAPs) for Rab7 inactivation (Stroupe 2018). Notably, by immunofluorescence staining we observed that nestin colocalizes more with TBC1D15 (Jongsma et al. 2020), a Rab7 GAP, but not other Rab7 GAPs or GEFs such as TBC1D2A (Frasa et al. 2012), TBC1D2B (Kanno et al. 2010), TBC1D5 (Jimenez‐Orgaz et al. 2018), or ccz1 (Wu et al. 2021) (Figure 6a and Figure S7a,b). To confirm whether nestin regulates Rab7 activity through TBC1D15, we knock down TBC1D15 in primary mouse lung fibroblasts using short hairpin RNAs. The results showed that TBC1D15 knockdown rescued Rab7 activity in Nestin‐knockdown cells (Figure 6b,c). Interestingly, MVBs colocalized more with Rab7 in Nestin‐knockdown cells than in control cells, which was blocked by TBC1D15 knockdown (Figure 6d,e). Consistently, TBC1D15 knockdown restored the levels of EVs markers CD63 and TSG101 and total EVs protein in Nestin‐knockdown cells (Figure 6f–i and Figure S7c). Taken together, these data suggest that nestin recruits TBC1D15 to inactivate Rab7, thereby controlling EVs secretion in vitro.

### 3.7 ML‐098 Attenuates Pulmonary Fibrosis in Experimental Mouse Models

**FIGURE 7:**
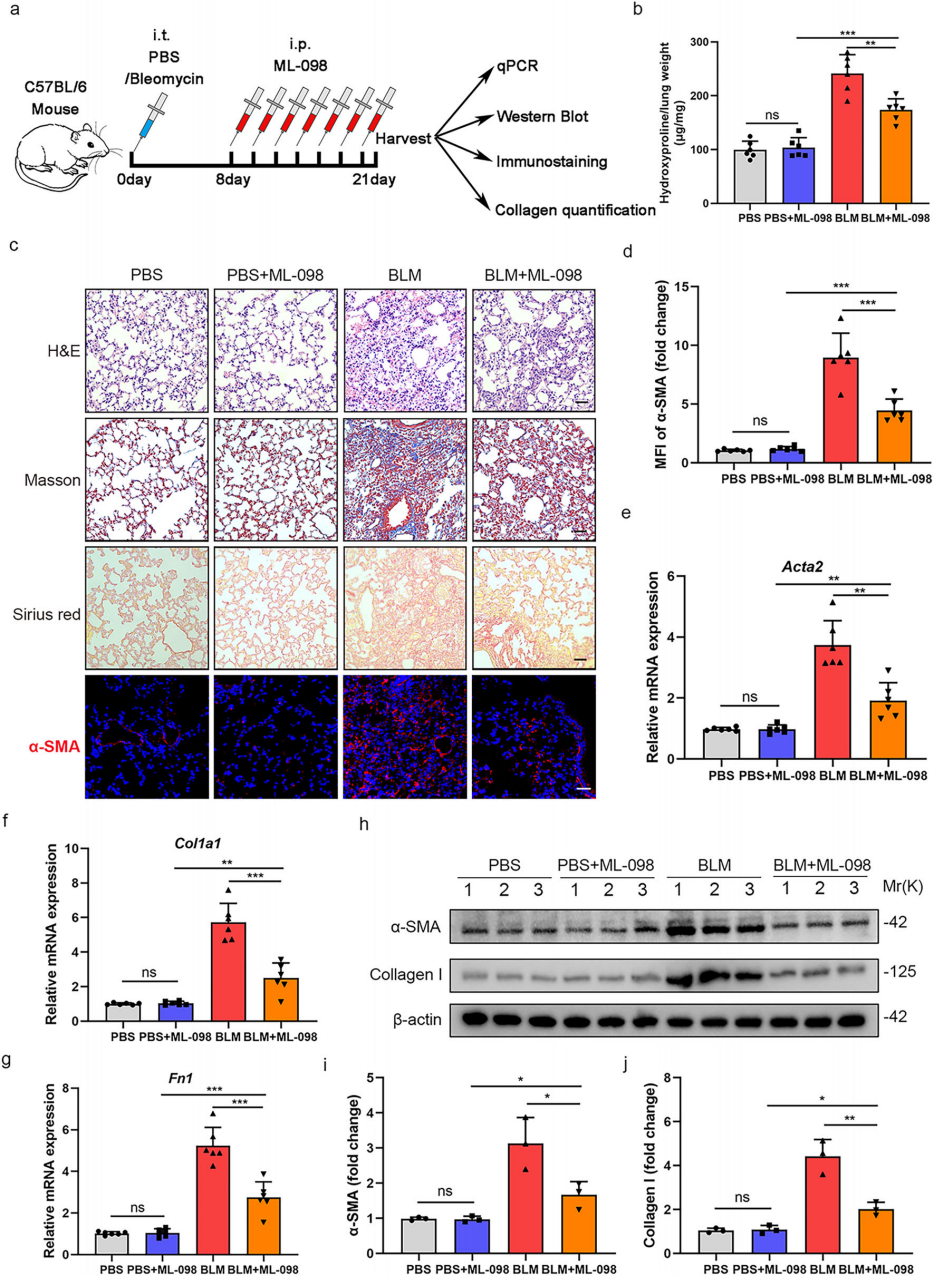
**ML‐098 attenuates pulmonary fibrosis in experimental mouse models. (**a) Experimental design. 8‐week‐old C57BL/6 mice were intratracheally injected with bleomycin (3U/kg) or PBS. Eight days later, mice were intraperitoneally delivered ML‐098 every other day. Samples were collected for analysis 21 days after bleomycin administration. Created in https://BioRender.com. (b) Hydroxyproline levels in lungs from C57/BL6 mice of the different groups (*n* = 6 per group). (c) Hematoxylin and eosin staining, Masson's trichrome staining, Sirius red staining and immunofluorescence images obtained using anti‐α‐SMA (red) antibody in lung from C57/BL6 mice of the different groups and (d) quantification analysis of α‐SMA from (c) (*n* = 6 per group). Scale bars = 100 µm. (e) qPCR analysis of Acta2 mRNA expression in the lungs of C57/BL6 mice from the different groups (*n* = 6 mice per group). (f) qPCR analysis of Col1a1 mRNA expression in the lungs of C57/BL6 mice from the different groups (*n* = 6 mice per group). (g) qPCR analysis of Fn1 mRNA expression in the lungs of C57/BL6 mice from the different groups (*n* = 6 mice per group). (h) Western blot and (i, j) quantification analysis of α‐SMA and collagen I expression in the lungs of C57/BL6 mice from the different groups (*n* = 3). Data are presented as the mean ± SD of three independent experiments; ns: no significance, *p < 0.05, **p < 0.01, ***p < 0.001; one‐way ANOVA and Tukey's multiple comparisons test.

Given that nestin promotes myofibroblast activation by recruiting TBC1D15 to inactivate Rab7 and regulating EVs secretion, we next examined the role of Rab7 in the pathogenesis of experimental pulmonary fibrosis in vivo. C57BL/6 mice were intratracheally injected with bleomycin or PBS, followed by intraperitoneal administration of ML‐098, a Rab7 activator, every other day (Figure 7a). At 21 days post‐bleomycin administration, lung tissues were harvested for analysis. ML‐098 treatment significantly attenuated bleomycin‐induced pulmonary fibrosis, as evidenced by reduced hydroxyproline levels and less severe inflammatory infiltration, interstitial collagen deposition, and fibrosis degree in lung samples in H&E, Masson's trichrome, and Sirius red staining (Figure 7b,c). Consistently, the expression levels of α‐SMA, collagen I, and fibronectin in lung tissues were markedly lower in the lungs of ML‐089‐treated mice compared to bleomycin‐treated controls (Figure 7d–j). These results demonstrate that Rab7 activation exerts a protective effect against pulmonary fibrosis in experimental mouse models.

## 4 Discussion

**FIGURE 8:**
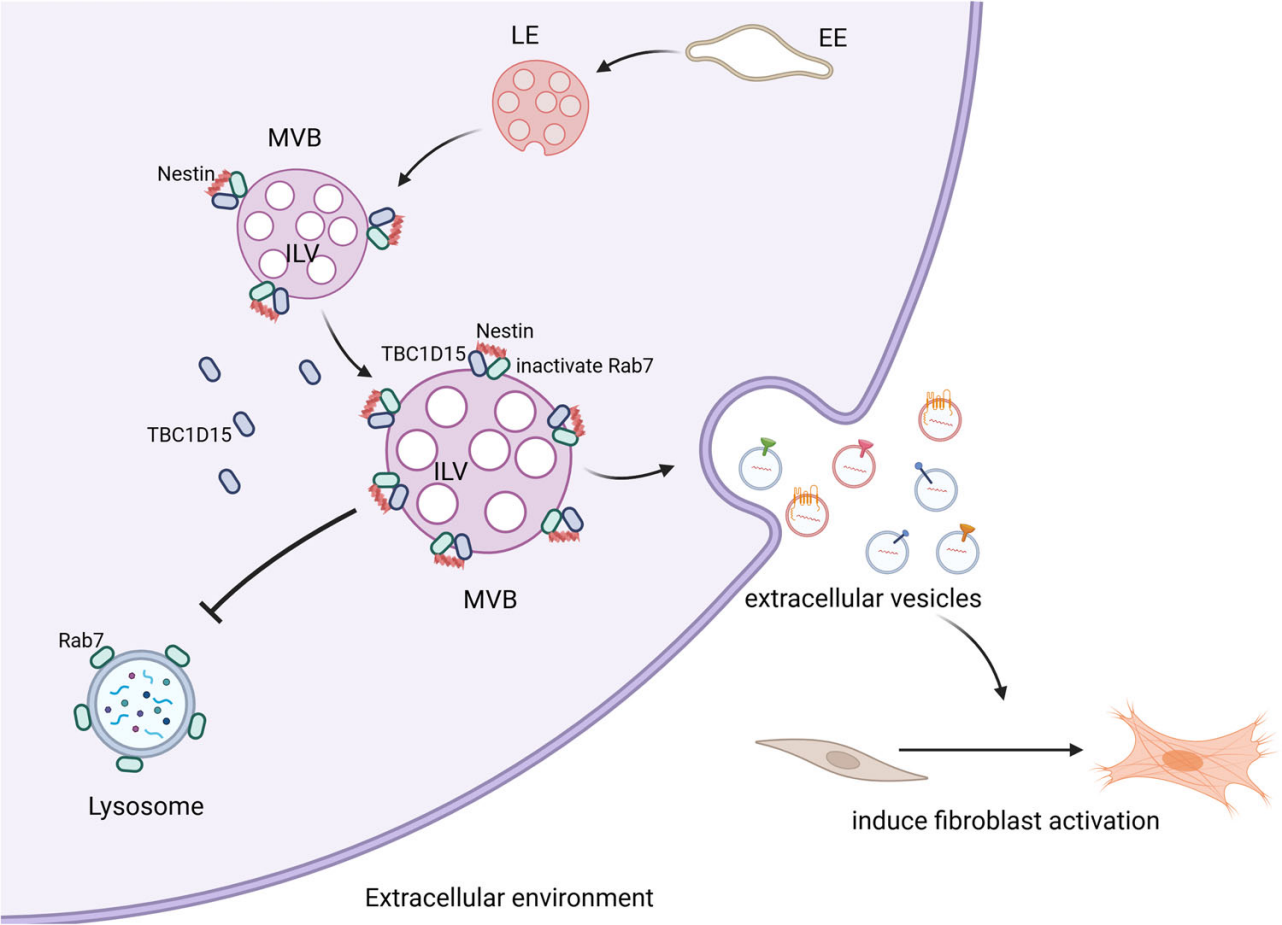
**Illustration of Nestin‐Rab7 axis in the regulation of EVs secretion in lung myofibroblasts within the lung microenvironment**. Nestin could inhibit the Rab7‐dependent degradation of multivesicular endosomes in lung myofibroblasts, thereby promoting the EVs secretion of lung myofibroblasts and profibrotic cascade amplification in pulmonary fibrosis. Created in https://BioRender.com.

IPF remains a deadly disease with limited therapeutic options, largely due to its complex and incompletely understood pathogenesis (Selman et al. 2001). We have previously established that the intermediate filament nestin is upregulated in lung myofibroblasts during pulmonary fibrosis, where it promotes progression by facilitating TβRI recycling (Wang et al. 2022). Here, we demonstrate a novel mechanism through which nestin drives fibrosis: it inhibits multivesicular body (MVB) degradation by inactivating Rab7 via TBC1D15, thereby promoting extracellular vesicle (EV) secretion. This nestin‐dependent increase in EV release amplifies the activation of lung myofibroblasts and disease progression. Collectively, our findings define a critical Nestin‐Rab7 axis that drives profibrotic cascade amplification within the lung microenvironment (Figure 8).

Beyond its established role as a marker of multipotent stem cells, nestin is significantly upregulated in the mesenchymal compartments of various fibrotic organs (Méndez‐Ferrer et al. 2010, Wang et al. 2022). A growing body of evidence has elucidated the molecular mechanisms by which nestin contributes to fibrosis progression. For example, in pulmonary fibrosis, nestin is upregulated in lung myofibroblasts and plays a key profibrogenic role by facilitating the Rab11‐dependent recycling of TβRI (Wang et al. 2022). Similarly, in liver fibrosis, TGF‐β induces nestin expression in hepatic stellate cells, creating a positive feedback loop that amplifies the TGFβ‐Smad2/3 pathway (Chen et al. 2021). In this study, we report a novel function for Nestin: it plays an essential role in driving EV secretion during the progression of pulmonary fibrosis. Given that nestin expression is tightly correlated with fibrosis, elucidating its mechanisms in regulating vesicle trafficking may reveal promising therapeutic targets for treating organ fibrosis.

Accumulating evidence indicates that EVs play a significant role in various pulmonary diseases, including acute lung injury, pulmonary fibrosis, and chronic obstructive pulmonary disease (Parimon et al. 2019). EVs can influence signaling networks in recipient cells by transferring bioactive cargo such as miRNAs, proteins, and lipids (Mathieu et al. 2019, Pegtel and Gould 2019, Kalluri and Lebleu 2020, Parimon et al. 2019). In the context of pulmonary fibrosis, EV levels are elevated in the bronchoalveolar lavage fluid (BALF) of both experimental models and IPF patients. For instance, EVs derived from lung fibroblasts can stimulate fibroblast proliferation, exerting a direct profibrogenic effect (Martin‐Medina et al. 2018). Furthermore, the key fibrogenic mediator TGF‐β1 induces nonsenescent fibroblasts to release increased numbers of EVs; these EVs promote an invasive phenotype in recipient fibroblasts via interactions between surface fibronectin and the α5β1 integrin receptor (Chanda et al. 2019). While these studies clearly document the profibrotic effects of EVs, the fundamental mechanisms that drive their heightened production and secretion in pulmonary fibrosis remain poorly understood. Our study addresses this gap by demonstrating that nestin interacts with Rab7 to inhibit MVB degradation and thereby promotes EV release from lung myofibroblasts, a process critically involved in amplifying the profibrotic cascade. Given the established importance of EVs in fibrosis, further exploration of the molecular mechanisms governing their secretion is warranted.

Rab GTPases, the largest family of membrane trafficking proteins in mammals, function as molecular switches that cycle between GTP‐bound active and GDP‐bound inactive states (Stenmark 2009). Several studies have shown that Rab GTPases are involved in regulating the functions of intermediate filament proteins. For example, it was reported that Rab43 downregulation significantly decreased the expression of vimentin, and Rab43 acts as a potential therapeutic target for glioma (Mruk et al. 2007). Similarly, Rab7a has been shown to regulate the function of vimentin through directly modulating its phosphorylation and assembly (Cogli et al. 2013). In addition, nestin had been shown to affect the migration of neural stem cells by regulating the activities of other small G proteins, RhoA and Rac1 (Yan et al. 2016). We previously reported that nestin binds to mitochondria‐related dynein (Drp1), leading to Cdk5 redistribution, mitochondrial remodeling, and the maintenance of NSPC stemness (Zhang et al. 2018). Moreover, nestin enhances TβRI recycling through the Rab11‐dependent pathway in lung myofibroblasts (Wang et al. 2022). In this study, we identified trimeric complexes containing Nestin, TBC1D15, and Rab7, and proved that nestin knockdown inhibits the secretion of EVs via Rab7. More importantly, we proved that the Rab7 activator ML‐098 exerts protective effects against pulmonary fibrosis in experimental mouse models. In the future, we plan to explore the relationship between nestin and the small G protein superfamily, which may provide an important theoretical basis for understanding how nestin regulates intracellular homeostasis.

EVs are recognized as key mediators of myofibroblast differentiation, carrying diverse bioactive molecules. For instance, EVs from IPF BALF promote the proliferation of primary human lung fibroblasts, a process primarily mediated by WNT5A (Martin‐Medina et al. 2018). In contrast, EVs derived from human bronchial epithelial cells can inhibit both myofibroblast differentiation and epithelial senescence through specific miRNAs that suppress WNT signaling (Kadota et al. 2021). Adding to this complexity, a multi‐omics analysis revealed that fibroblast‐secreted frizzled‐related protein 1 promotes lung fibrosis in vivo (Burgy et al. 2024). While these studies highlight the functional diversity of EV cargo, the mechanisms regulating EV quantity in fibrosis remain less explored. In the present study, we demonstrate that nestin increases EV secretion by inhibiting MVB degradation via TBC1D15‐mediated Rab7 inactivation, thereby promoting myofibroblast activation and pulmonary fibrosis. Furthermore, to identify specific profibrotic factors, we performed a proteomic analysis of EVs from lung fibroblasts treated with or without TGF‐β (Figure S8a,b). The results reveal a suite of differentially expressed proteins, leading us to hypothesize that the profibrotic effect of these EVs is not attributable to a single factor, but rather to the combined action of multiple proteins and potentially other cargo. The definitive identification of these key effector molecules is a primary objective of our future research.

While targeting nestin holds therapeutic promise for pulmonary fibrosis, its expression in a broad spectrum of progenitor and structural cells—including myofibroblasts, pericytes, and smooth muscle cells—warrants careful consideration of potential systemic and off‐target effects. Inhibition could theoretically interfere with lung regeneration and disrupt physiological functions in other organs. To mitigate these risks and enhance translational applicability, we propose several strategic approaches. First, utilizing localized pulmonary administration (precision delivery), such as inhaled nanoscale formulations, could maximize on‐target efficacy in the lungs while minimizing systemic exposure. Second, a dynamic dosing regimen, involving higher‐dose intervention during the acute fibrotic phase followed by lower‐dose maintenance, could help balance efficacy with reduced long‐term side effects.

Last, combination therapy employing nestin inhibition as part of a synergistic regimen with standard‐of‐care antifibrotics could allow for lower, safer doses of each agent while achieving superior outcomes. Future work will prioritize in‐depth preclinical validation, including long‐term safety and toxicology studies in advanced animal models, to thoroughly evaluate these strategies before clinical translation. In conclusion, our findings reveal a role by which nestin modulates the TBC1D15‐mediated Rab7 inactivation and EVs secretion in lung myofibroblasts. We propose that the Nestin‐Rab7 axis promotes the EVs secretion of myofibroblasts in the lung microenvironment, leading to an amplification of profibrotic responses. Understanding the mechanisms that control changes in the lung microenvironment may offer promising therapeutic strategies to ameliorate or even reverse pulmonary fibrosis.

## Author Contributions

**Xiaofan Lai**: conceptualization, writing – original draft, investigation, project administration, supervision. **Yong Xiao**: writing – original draft, conceptualization, investigation. **Yingying Lin**: methodology, project administration, investigation. **Senyu Yao**: investigation, methodology, software. **Bin Wang**: investigation, methodology, writing – review and editing. **Hainan Chen**: investigation, methodology. **Tianxiang Lei**: project administration, investigation. **Shaojie Huang**: investigation, formal analysis. **Chenxing Lei**: investigation, formal analysis. **Qihao Zeng**: validation, visualization. **Yuan Qiu**: methodology, project administration. **Hong Chen**: formal analysis. **Tao Wang**: supervision. **Jiancheng Wang**: writing – review and editing, supervision, conceptualization, funding acquisition. **Andy Peng Xiang**: conceptualization, funding acquisition, writing – review and editing, data curation.

## Funding

This work was supported by National Natural Science Foundation of China (82200073, 82400079, 32170799,32130046, 82371608, 82430050, 82371611, 82371609, 82301847, 82171617, 82301796, 82504017), National Key Research and Development Program of China (2021YFA1100601, 2022YFA1104100),  Natural Science Foundation of Guangdong Province (2022A1515010371), Guangdong Basic and Applied Basic Research Foundation (2023B1515020016), Key Scientific and Technological Program of Guangzhou City (2023B01J1002), Sanming Project of Medicine in Shenzhen Nanshan (SZSM202103012), China Post‐doctoral Science Foundation (2025M772123), Shenzhen Science and Technology Program (JCYJ20240813150417024).

## Conflicts of Interest

The authors declare no conflicts of interest.

## Supporting information




**Supplementary Material**: jev270223‐sup‐0001‐SuppMat.docx

## Data Availability

The authors have nothing to report.
