# Supplementary material for: Myofibroblast‐Derived Extracellular Vesicles Drive Profibrotic Cascade Amplification in Pulmonary Fibrosis via the Nestin‐Rab7 Axis
Source: J Extracell Vesicles. 2026 Jan 6;15(1):e70223. doi: 10.1002/jev2.70223 (PMC12775576; doi:10.1002/jev2.70223)
Supplement: Supplementary file 1 — Supplementary Material: jev270223‐sup‐0001‐SuppMat.docx [file JEV2-15-e70223-s001.docx]

**Supplementary Materials
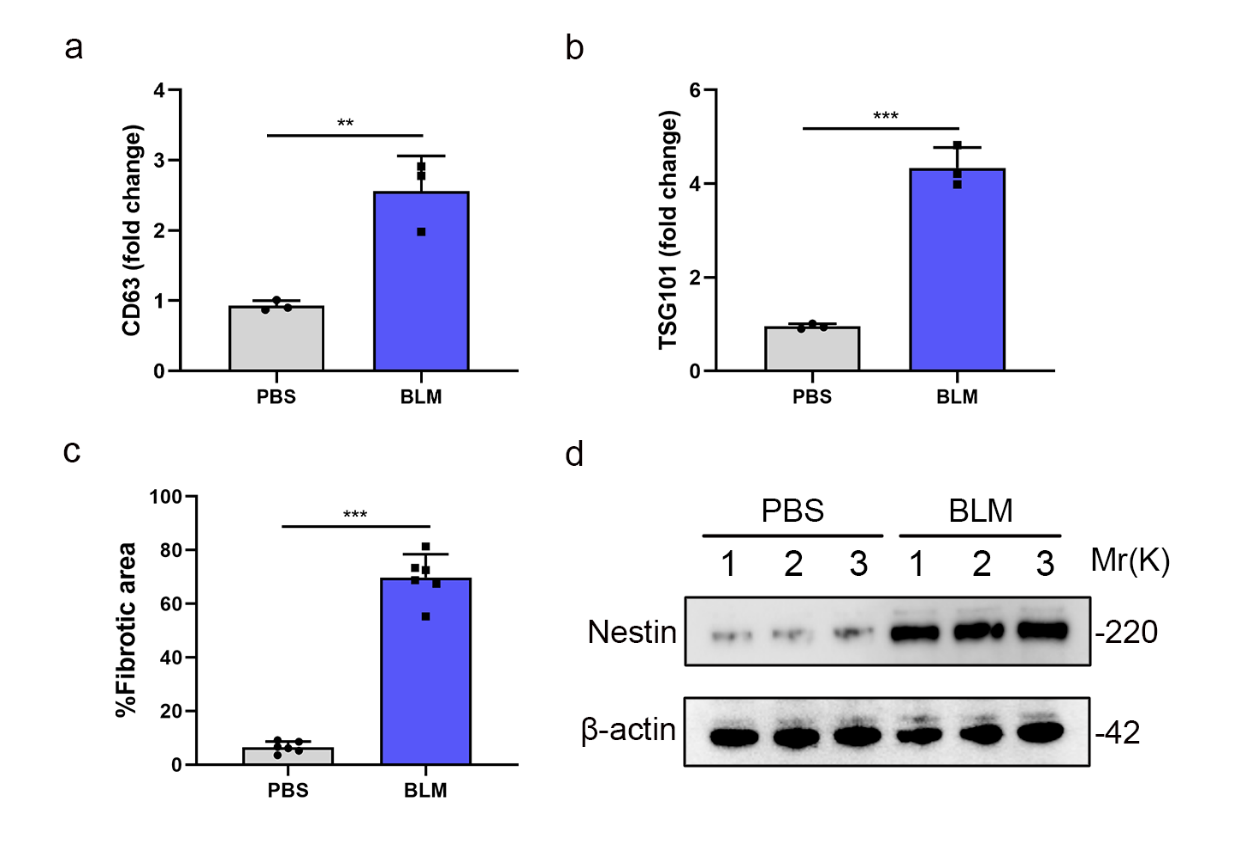
Supplementary figure 1:**

a) Quantification analysis of CD63 expression in Figure 1b. b) Quantification analysis of TSG101 expression in Figure 1b. c) Quantification analysis of fibrotic area from Masson’s trichrome staining in Figure 1e. d) Western blot analysis of Nestin expression in the lungs of C57/BL6 mice from the different groups. Data are presented as the mean ± SD of three independent experiments; **P<0.01; ***P<0.001; One-way ANOVA and Tukey’s multiple comparisons test.


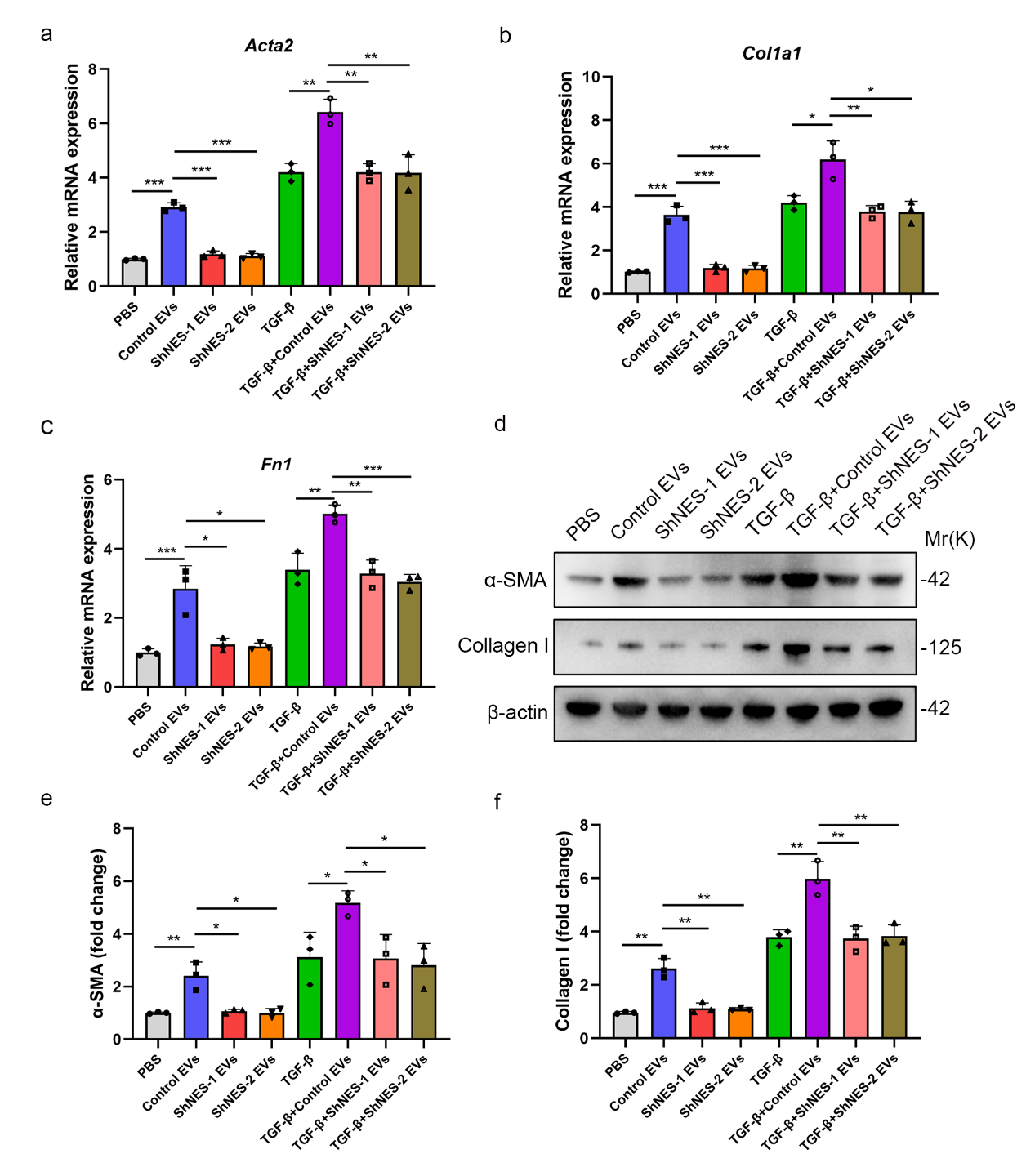


**Supplementary figure 2:**

a) qPCR analysis of Acta2 mRNA expression in primary mouse lung fibroblasts treated with EVs with or without TGF-β (5 ng·mL^−1^) in different groups for 72 h. b) qPCR analysis of Col1a1 mRNA expression in primary mouse lung fibroblasts treated with EVs with or without TGF-β (5 ng·mL^−1^) in different groups for 72 h. c) qPCR analysis of Fn1 mRNA expression in primary mouse lung fibroblasts treated with EVs with or without TGF-β (5 ng·mL^−1^) in different groups for 72 h. d) Western blot and e, f) quantification analysis of α-SMA and Collagen I expression in the lungs of C57/BL6 mice from the different groups. Data are presented as the mean ± SD of three independent experiments; *P<0.05；**P<0.01；***P<0.001; One-way ANOVA and Tukey’s multiple comparisons test.


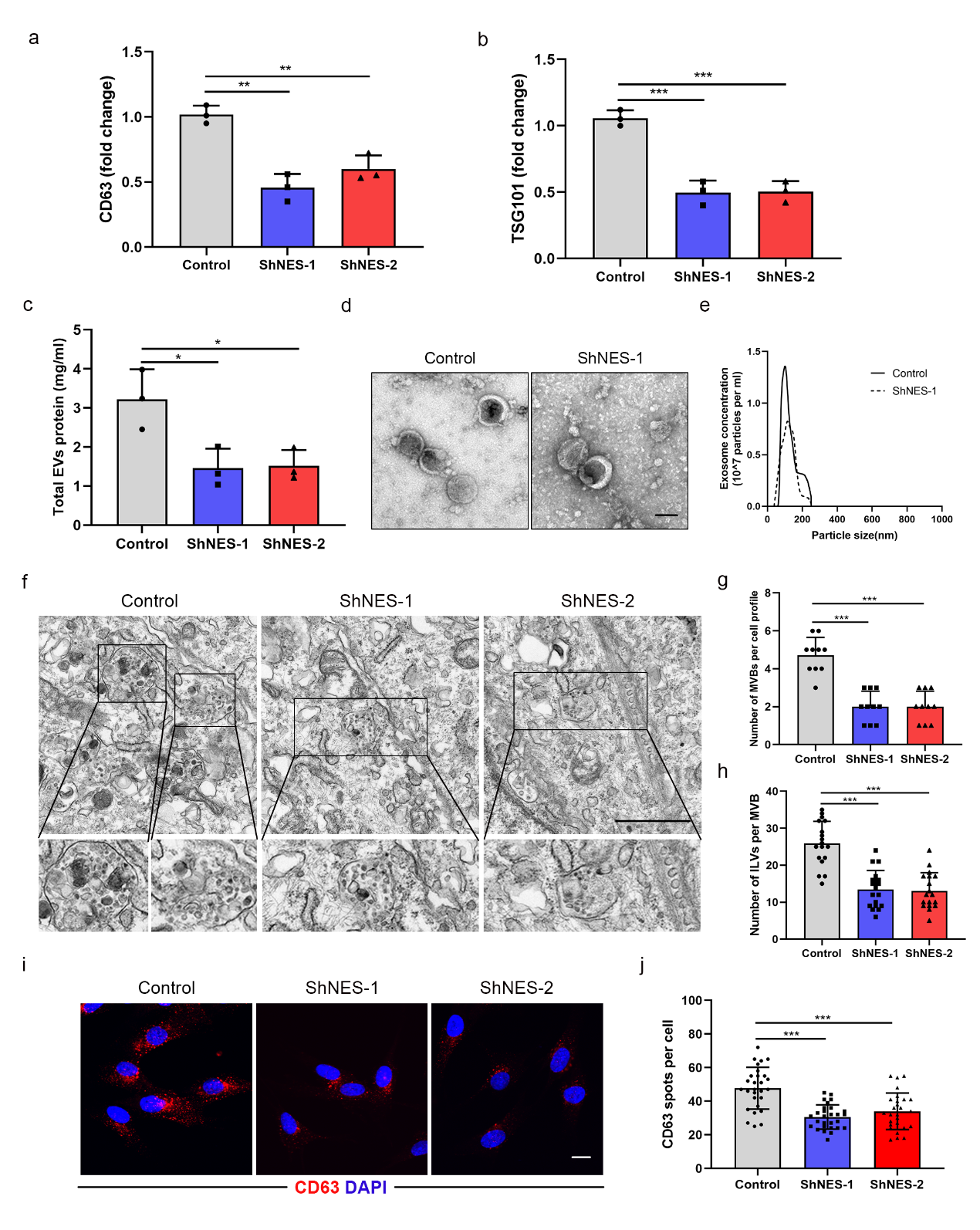


**Supplementary figure 3:**

a) Quantification analysis of CD63 expression in Figure 4c. b) Quantification analysis of TSG101 expression in Figure 4c. c) Concentrations of EVs proteins in cell culture supernatants of Nestin-knockdown cells and control cells isolated by serial ultracentrifugation. d) Representative electron microscopic images of EVs purified from cell culture supernatants of Nestin-knockdown cells and control cells. Scale bar = 100 nm. c) Representative NTA traces of EVs derived from Nestin-knockdown cells and control cells. f) Representative electron microscopic images of Nestin-knockdown cells and control cells. Scale bar = 1 μm. g) The number of MVBs per cell profile. h) The number of ILVs per MVB. i) Immunofluorescence staining and j) quantification analysis of Nestin-knockdown cells and control cells using anti-CD63 (red) antibody. Scale bars: 20 µm. Data are presented as the mean ± SD of three independent experiments; *P<0.05, **P<0.01, ***P<0.001.


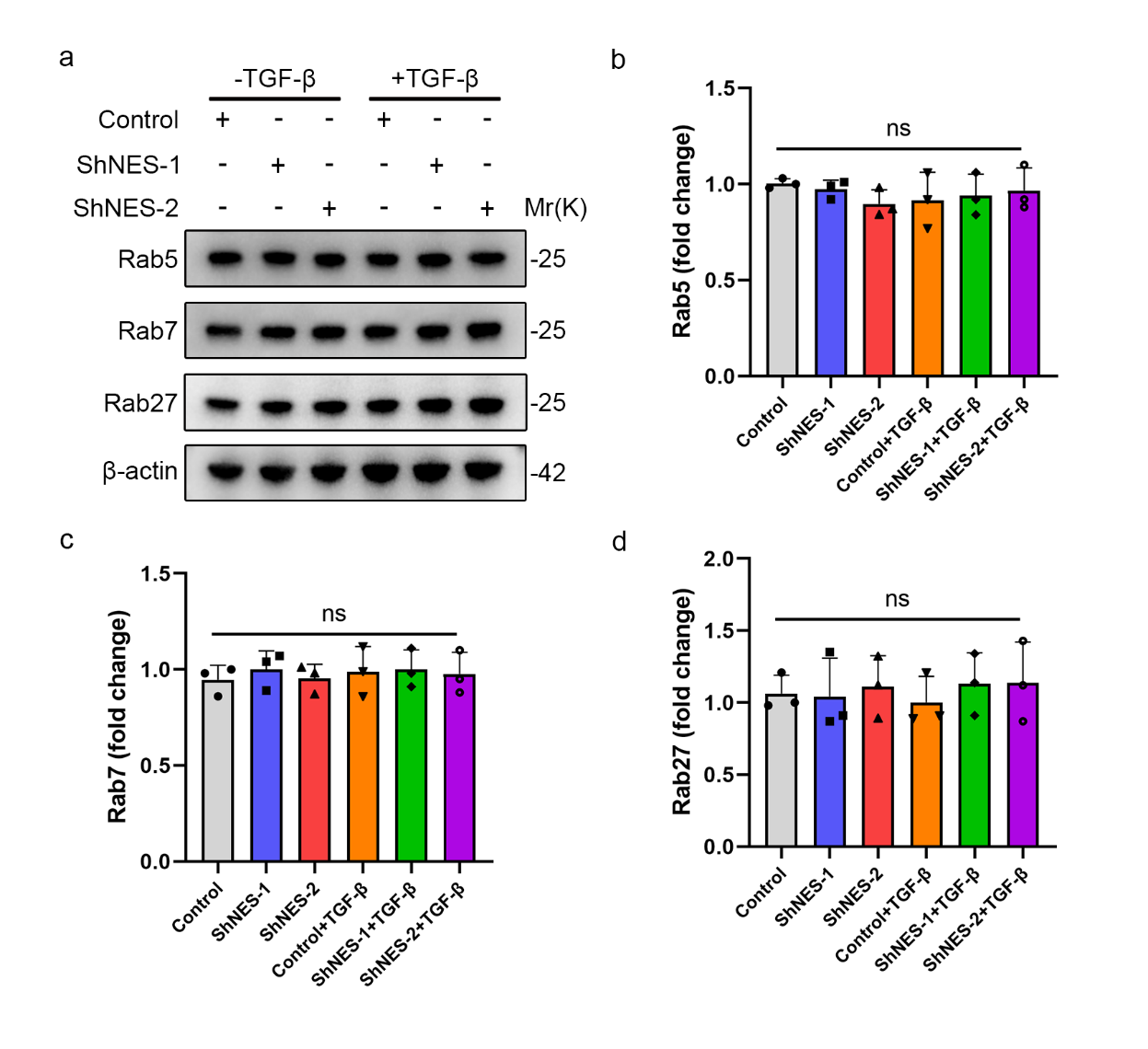


**Supplementary figure 4:**

a) Western blot and b-d) quantification analysis of Rab5, Rab7 and Rab27 expression in Nestin-knockdown cells and control cells treated with or without TGF-β (5 ng·mL^−1^). Data are presented as the mean ± SD; ns: no significance; One-way ANOVA and Tukey’s multiple comparisons test.

**
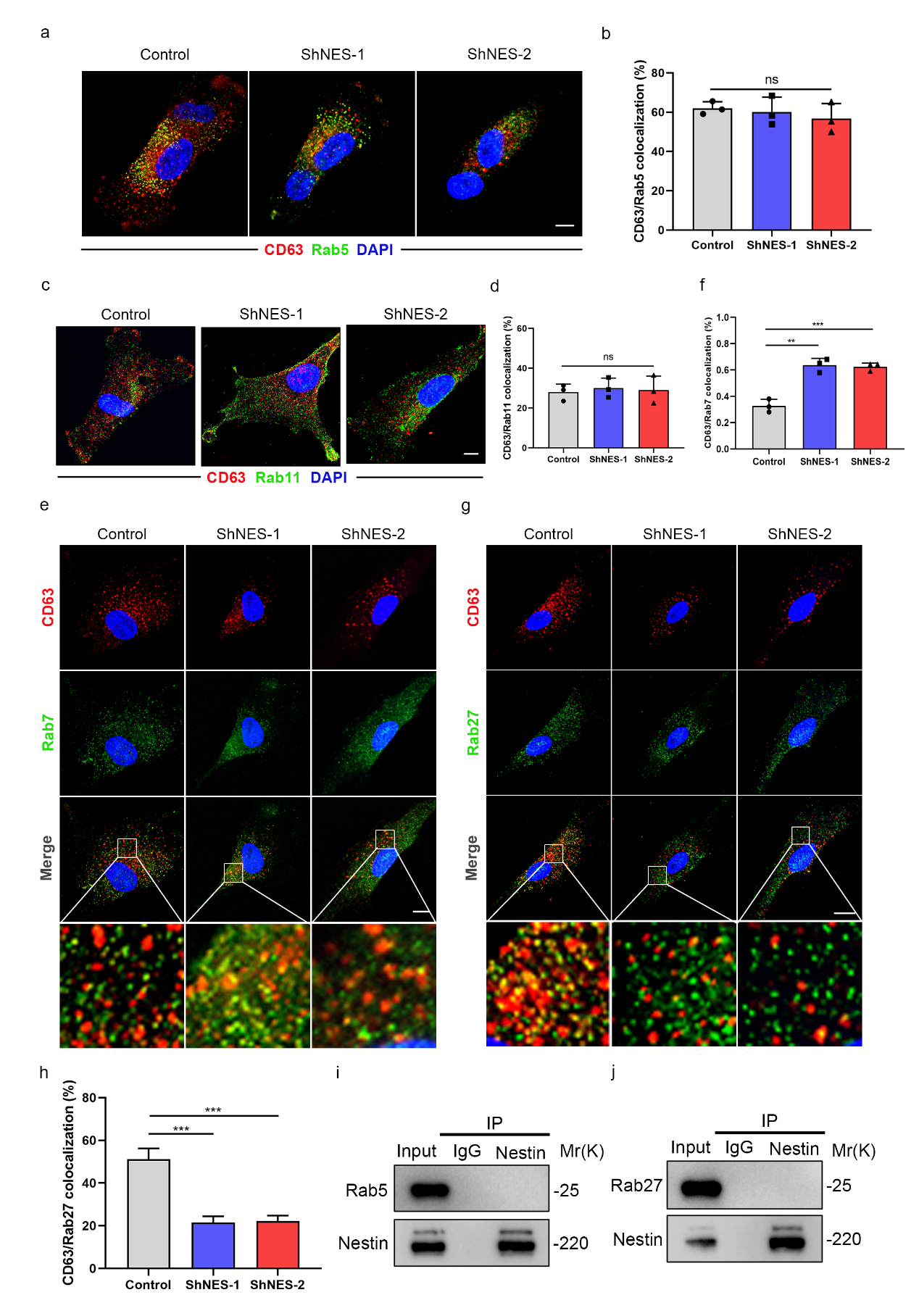
**

**Supplementary figure 5:**

a) Immunofluorescence staining and b) colocalization analysis of Nestin-knockdown cells and control cells treated with TGF-β (5 ng·mL^−1^) using anti-CD63 (red) and anti-Rab5 (green) antibody. Scale bars: 10 µm. c) Immunofluorescence staining d) colocalization analysis of primary mouse lung fibroblasts using anti-CD63 (red) and anti-Rab11 (green) antibody. Scale bars: 10 µm. e) Immunofluorescence staining and f) colocalization analysis of Nestin-knockdown cells and control cells treated with TGF-β (5 ng·mL^−1^) using anti-CD63 (red) and anti-Rab7 (green) antibody. Scale bars: 20 µm. g) Immunofluorescence staining and h) colocalization analysis of Nestin-knockdown cells and control cells treated with TGF-β (5 ng·mL^−1^) using anti-CD63 (red) and anti-Rab27 (green) antibody. Scale bars: 20 µm. i) Immunoprecipitation was performed using an anti-Nestin antibody, and immunoblotting of the protein levels of Rab5 in primary mouse lung fibroblasts. j) Immunoprecipitation was performed using an anti-Nestin antibody, and immunoblotting of the protein levels of Rab27 in primary mouse lung fibroblasts. Data are presented as the mean ± SD; ns: no significance; **P<0.01, ***P<0.001; One-way ANOVA and Tukey’s multiple comparisons test.


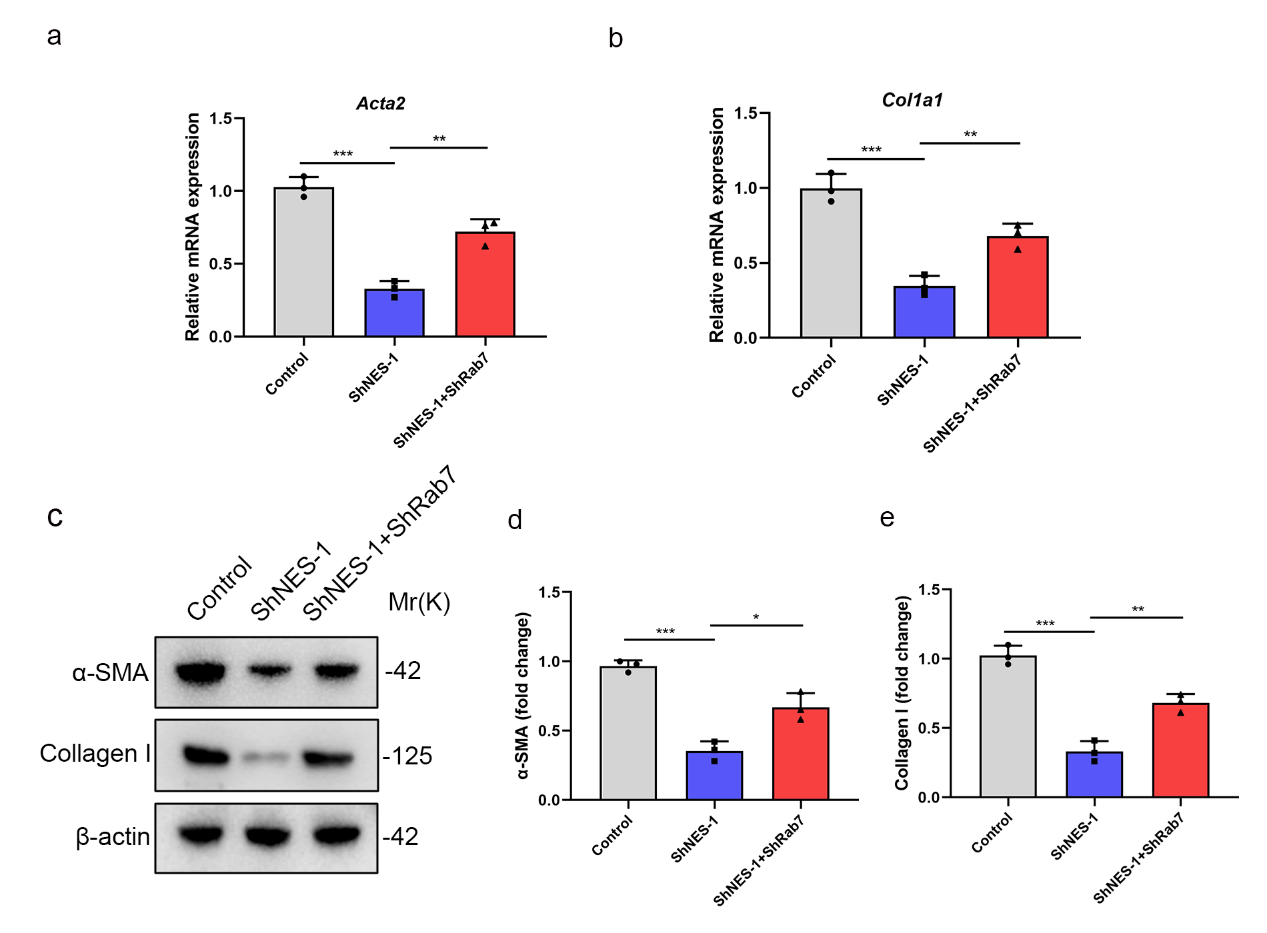


**Supplementary figure 6:**

a) qPCR analysis of Acta2 mRNA expression in primary mouse lung fibroblasts with Nestin knockdown and Rab7 knockdown after TGF-β (5 ng·mL^−1^) treatment. b) qPCR analysis of Col1a1 mRNA expression in primary mouse lung fibroblasts with Nestin knockdown and Rab7 knockdown after TGF-β (5 ng·mL^−1^) treatment. c) Western blot and d-e) quantification analysis of α-SMA and Collagen I expression in primary mouse lung fibroblasts with Nestin knockdown and Rab7 knockdown after TGF-β (5 ng·mL^−1^) treatment. Data are presented as the mean ± SD; ns: no significance; *P<0.05, **P<0.01, ***P<0.001; One-way ANOVA and Tukey’s multiple comparisons test.

**
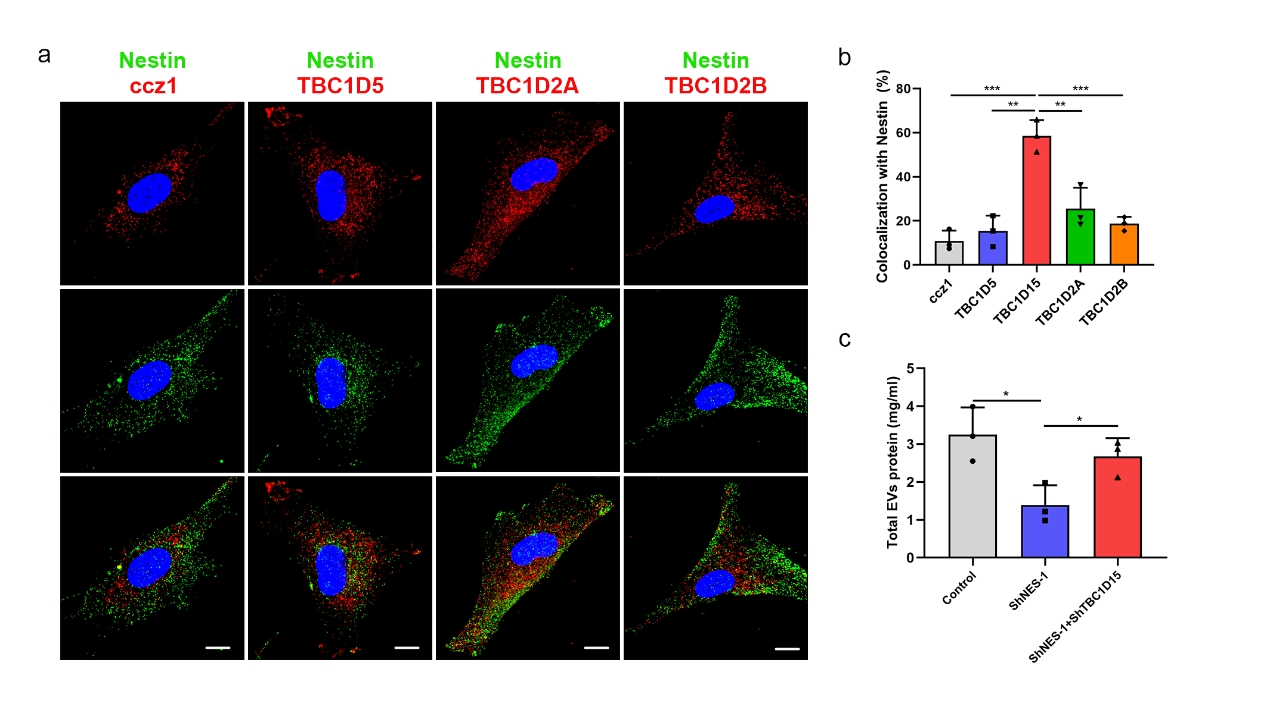
Supplementary figure 7:**

a) Immunofluorescence staining and b) colocalization analysis of primary mouse lung fibroblasts using anti-Nestin (green) and anti-ccz1, TBC1D5, TBC1D2A or TBC1D2B (red) antibody. Scale bars: 20 µm. c) Concentrations of EVs proteins purified by serial ultracentrifugation from cell culture supernatants from equal numbers of Nestin-knockdown cells and control cells treated with or without TBC1D15 knockdown. Data are presented as the mean ± SD; *P<0.05, **P<0.01, ***P<0.001; One-way ANOVA and Tukey’s multiple comparisons test.


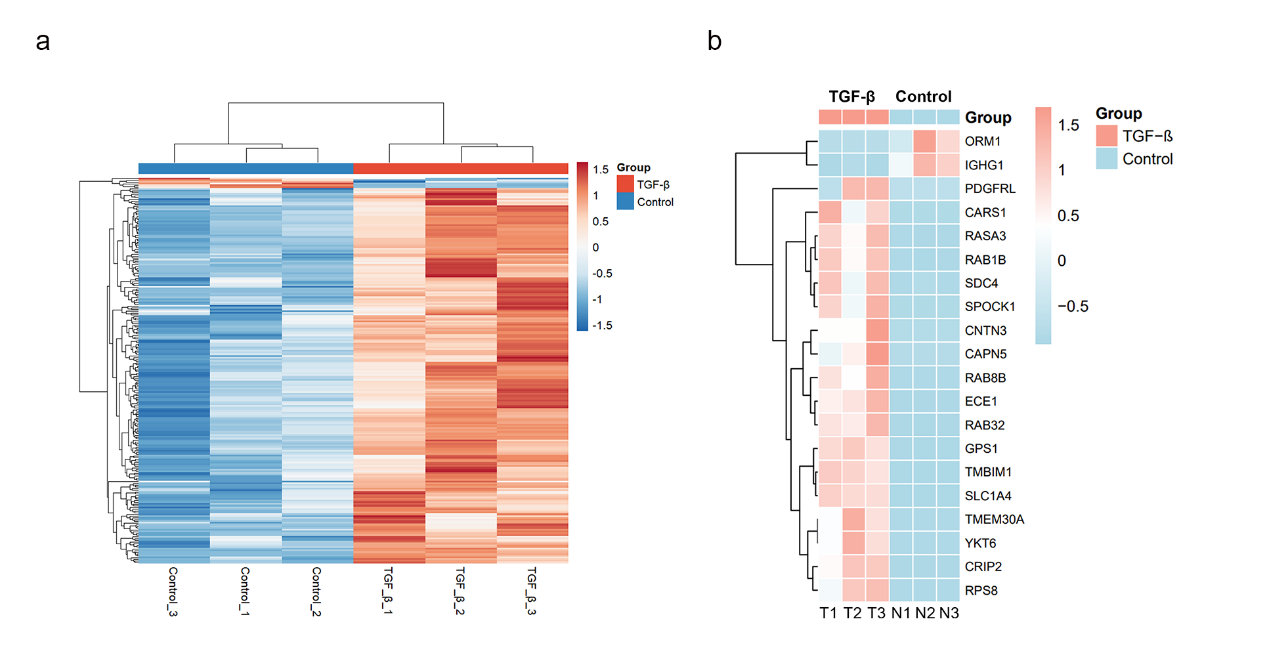


**Supplementary figure 8:**

a) Clustering analysis of the global expression profile from protein sequencing of EVs from fibroblasts treated with or without TGF-β. b) The expression of the top 20 protein genes with the most significant differences in 6 samples.

**Table S1: Target sequences of shRNAs used.**

| **Name** | **Sequences (5′ to 3′)** |
| --- | --- |
| **Human：**  **NESTIN shRNA#1** | **5′-GCTAGTCCCTGCCTGAATA-3′** |
| **NESTIN shRNA#2**  **Mouse：** | **5′-GCAGACATCATTGGTGTTAAT-3′** |
| **NESTIN shRNA1** | **5′-GGAAGAAGTTCCCAGGCTTCT-3′** |
| **NESTIN shRNA2** | **5′-GCTGAAGCTGCATTTCCTTGG-3′** |
| **NESTIN shRNA（AAV-6）**  **Scramble shRNA（AAV-6）** | **5′-GTGAGACTCTGGAATGCAA-3′**  **5′-TTCTCCGAACGTGTCACGTAA-3′** |

**Table S2: Primer used to amplify the human transcripts or genome DNA during PCR.** Related to Experimental Procedures.

| **Gene** | **Sequences (5′ to 3′)** | **application** |
| --- | --- | --- |
| **h*NESTIN***  **h*GAPDH*** | **Forward: 5’-CTGCTACCCTTGAGACACCTG-3’**  **Reverse: 5’-GGGCTCTGATCTCTGCATCTAC-3’**  **Forward: 5’-GTCGGAGTCAACGGATTT-3’**  **Reverse:5’-GGAATCATATTGGAACATGTAAACC-3’** | **qPCR**  **qPCR** |
| **m*NESTIN*** | **Forward: 5’-GCAGGAGAAGCAGGGTCTAC-3’**  **Reverse: 5’-GGGGTCAGGAAAGCCAA-3’** | **qPCR** |
| **m*Acta2*** | **Forward: 5’-TGAGACCTTCAATGTCCCCGC-3’**  **Reverse: 5’-TCACACCATCTCCAGAGTCCAGC-3’** | **qPCR** |
| **m*Fn1***  **m*col1a1*** | **Forward: 5’-GGTGTAGCACAACTTCCAATTACG-3’**  **Reverse: 5’-GGAATTTCCGCCTCGAGTCT-3’**  **Forward:** **5’-TGACTGGAAGAGCGGAGAGT-3’**  **Reverse:** **5’-GTTCGGGCTGATGTACCAGT-3’** | **qPCR**  **qPCR** |
| **m*18S*** | **Forward: 5’-GTAACCCGTTGAACCCCATT-3’**  **Reverse: 5’-CCATCCAATCGGTAGTAGCG-3’** | **qPCR** |

**Table S3: Primary and secondary antibodies.**

| **Product** | **Catalogue Number** | **Supplier** |
| --- | --- | --- |
| **Primary antibody:** |  |  |
| **WB and IP:** |  |  |
| **mouse anti-Nestin**  **mouse anti-Nestin** | **611658**  **MAB353** | **BD Biosciences**  **Millipore** |
| **rabbit anti-DYKDDDDK（Flag Tag）**  **mouse anti-DYKDDDDK（Flag Tag）**  **mouse Anti-β-actin** | **14793**  **8146**  **600081** | **Cell Signaling Technology**  **Cell Signaling Technology**  **Proteintech** |
| **rabbit anti-Collagen I** | **ab34710** | **Abcam** |
| **mouse anti-α-SMA** | **ab7817** | **Abcam** |
| **mouse anti-Rab7**  **rabbit anti-Rab7** | **sc-376362**  **9367** | **Santa Cruz Biotechnology**  **Cell Signaling Technology** |
| **mouse anti-Rab5**  **rabbit anti-Rab27**  **mouse anti-CD63**  **rabbit anti-CD63**  **mouse anti-CD63**  **rabbit anti-TSG101** | **sc-46692**  **69295**  **ab59479**  **25682-1-AP**  **67605-1-Ig**  **ab125011** | **Santa Cruz Biotechnology**  **Cell Signaling Technology**  **Abcam**  **Proteintech**  **Proteintech**  **Abcam** |
| **IF:** |  |  |
| **rabbit anti-Nestin** | **ABD69** | **Millipore** |
| **mouse anti-Nestin**  **mouse anti-Nestin** | **MAB5326**  **MAB353** | **Millipore**  **Millipore** |
| **rabbit anti-CD31**  **rabbit anti-CD31** | **ab28364**  **ab76533** | **Abcam**  **Abcam** |
| **mouse anti-α-SMA** | **ab7817** | **Abcam** |
| **rabbit anti-NG2**  **rabbit anti-NG2** | **AB5320**  **ab183929** | **Millipore**  **Abcam** |
| **rabbit anti-Prosurfactant Protein C**  **mouse anti-Rab5**  **rabbit anti-Rab27**  **mouse anti-Rab7**  **rabbit anti-Rab7** | **AB3786**  **sc-46692**  **69295**  **sc-376362**  **9367** | **Millipore**  **Santa Cruz Biotechnology**  **Cell Signaling Technology**  **Santa Cruz Biotechnology**  **Cell Signaling Technology** |
| **mouse anti-Aquaporin 5**  **rabbit anti-Aquaporin 5**  **rabbit anti-Calponin 1**  **mouse anti-CD63**  **mouse anti-CD63**  **rabbit anti-TSG101**  **rabbit anti-CD63**  **mouse anti-CD63**  **mouse anti-CCZ1**  **mouse anti-TBC1D2B**  **mouse anti-TBC1D5**  **rabbit anti-TBC1D2**  **rabbit anti-TBC1D15** | **sc-514022**  **ab92320**  **ab46794**  **ab59479**  **ab8219**  **ab125011**  **25682-1-AP**  **67605-1-Ig**  **sc-514290**  **sc-398906**  **sc-376296**  **204304-T32-50**  **CSB-PA823452LA01HU-1** | **Santa Cruz Biotechnology**  **Abcam**  **Abcam**  **Abcam**  **Abcam**  **Abcam**  **Proteintech**  **Proteintech**  **Santa Cruz Biotechnology**  **Santa Cruz Biotechnology**  **Santa Cruz Biotechnology**  **Sino Biological**  **CUSABIO** |
| **Secondary antibody:**  **WB:**  **anti-mouse IgG HRP-linked Ab**  **anti-rabbit IgG HRP-linked Ab**  **IF:**  **goat anti-mouse IgG Alexa 488**  **goat anti-rabbit IgG Alexa 488**  **goat anti-rabbit IgG Alexa 555**  **goat anti-mouse IgG Alexa 555**  **goat anti-rat IgG Alexa 488**  **goat anti-rat IgG Alexa 555**  **goat anti-rat IgG Alexa 647**  **goat anti-rabbit IgG Alexa 647** | **7076**  **7074**  **A11001**  **A11008**  **A21428**  **A21422**  **A11006**  **A21434**  **A-21235**  **A-20991** | **Cell Signaling Technology**  **Cell Signaling Technology**  **Invitrogen**  **Invitrogen**  **Invitrogen**  **Invitrogen**  **Invitrogen**  **Invitrogen**  **Invitrogen**  **Invitrogen** |
